# Associations between inflammatory-lipid indices and clinical outcomes in ischemic stroke patients treated with mechanical thrombectomy

**DOI:** 10.3389/fnagi.2026.1919645

**Published:** 2026-10-02

**Authors:** Ao Qian, Jia Duan, Wenli Xing, Longyi Zheng, Shuang Tang

**Affiliations:** 1 Department of Cerebrovascular Disease, Suining Central Hospital, Suining, Sichuan, China; 2 Department of Radiology, Suining Central Hospital, Suining, Sichuan, China

**Keywords:** acute ischemic stroke, inflammation, large vessel occlusion, lipid, mechanical thrombectomy, outcomes

## Abstract

**Purpose:**

Inflammation and dyslipidemia are critical in post-stroke prognosis. We conducted a comprehensive analysis of six common inflammatory-lipid indices to evaluate the associations between these indices and four clinical outcomes after mechanical thrombectomy (MT) for acute ischemic stroke (AIS), and to identify generalizable consistently associated markers for all four clinical outcomes.

**Methods:**

In this retrospective studyassociations between inflammatory-lipid indices, namely monocyte-to-high-density lipoprotein cholesterol ratio (MHR), platelet-to-HDL-C ratio (PHR), neutrophil-to-HDL-C (NHR), lymphocyte-to-HDL-C ratio (LHR), fibrinogen-to-HDL-C ratio (FHR), and C-reactive protein-to-HDL-C ratio (CRP/HDL-C), and four post-MT clinical outcomes comprised hemorrhagic transformation (HT), malignant cerebral edema (MCE) 90-day unfavorable outcome and all-cause mortality were evaluated using multivariable analyses. False discovery rate (FDR) correction was applied for multiple comparisons. NHR, which demonstrated consistent significance for all four clinical outcomes, was subjected to further analyses.

**Results:**

A total of 513 patients were included., demonstrated The incidences of HT, MCE, unfavorable outcome, and all-cause mortality were 30.6% (157), 22.6% (116), 54.8% (281), and 25.9% (133), respectively. The NHR was significantly associated with all four clinical outcomes (HT: odds ratio [OR] 1.079, 95% confidence interval [CI] 1.023–1.142, p-FDR = 0.034; MCE: OR 1.089, 95% CI 1.031–1.154, p-FDR = 0.008; unfavorable outcome: OR 1.177, 95% CI 1.098–1.269, p-FDR < 0.001; all-cause mortality: hazard ratio [HR] 1.047, 95% CI 1.012–1.084, p-FDR = 0.022). In the subgroup analysis for all-cause mortality, an interaction between NHR and TOAST classification was observed (*p* for interaction = 0.023), indicating that the association with all-cause mortality was pronounced in patients with cardioembolic stroke (HR 1.109, 95% CI 1.055–1.165). Mediation analysis showed that HT (23.9%, *p* < 0.001) and MCE (23.8%, *p* < 0.001) statistically accounted for part of the associations between NHR and unfavorable outcome. The relationship between NHR and unfavorable outcome may be partially mediated by HT (23.9%, *p* < 0.001) and MCE (23.8%, *p* < 0.001).

**Conclusion:**

NHR may serve as a potentially useful complementary marker of post-MT HT, MCE, unfavorable outcome, and all-cause mortality. Its association with all-cause mortality appears stronger in patients with cardioembolic stroke. HT and MCE may act as mediators linking NHR to unfavorable outcomes. External validation is required to verify these findings.

## 1 Introduction

With the progression of global population aging, acute ischemic stroke (AIS) has become a major life-threatening disease worldwide. Acute large vessel occlusion (LVO), the most severe subtype of AIS, is characterized by high disability and mortality, imposing substantial burdens on individuals and society (Saini et al., 2021; Herpich and Rincon, 2020). Timely and effective reperfusion therapy is essential to improve outcomes in patients with AIS (Lattanzi et al., 2021). In recent decades, advances in endovascular technologyhave established mechanical thrombectomy (MT) as the standard treatment for acute LVO, enabling rapid recanalization, salvage of the ischemic penumbra, and reduction of cerebral injury (Huang et al., 2019a). However, early complications and adverse outcomes remain persistent challenges post-MT for acute LVO. Hemorrhagic transformation (HT) and cerebral edema are common post-MT complications. Severe HT and malignant cerebral edema (MCE) can cause rapid neurological deterioration, midline shift, brain herniation, and even death (Huang et al., 2019b). Moreover, even following standardized therapy, approximately 50–70% of patients fail to achieve functional independence (Zeng et al., 2022; Lattanzi et al., 2021; Chen et al., 2024; Wang et al., 2024; Tang et al., 2023). Therefore, identifying factors associated with early complications and long-term clinical outcomes after MT may help refine therapeutic strategies and improve patient prognosis.

Inflammation and dyslipidemia play critical roles in the occurrence and progression of stroke (Puig et al., 2020; van Dam-Nolen et al., 2021). Previous reports have confirmed the correlation of inflammatory and lipid parameters with the prognosis of patients with ischemic stroke (Duan et al., 2025; Qian et al., 2024). In a prospective study, Fakhari et al. identified the neutrophil count and neutrophil-to-lymphocyte ratio as inflammatory biomarkers of functional outcomes in patients with AIS (Fakhari et al., 2024). Furthermore, lipid-derived indices, such as the atherogenic index of plasma, have also been found to be correlated with short-term mortality and unfavorable outcome in patients with ischemic stroke (Tatar et al., 2025; Duan et al., 2025). Recent studies have proposed that composite inflammatory-lipid indices, derived from peripheral blood parameters, are associated with adverse outcomes following MT for AIS (Li et al., 2021; Xu et al., 2026). However, most of these studies have focused on single indicators or specific clinical endpoints. For example, Ci et al. demonstrated an association between the neutrophil-to-high-density lipoprotein cholesterol ratio (NHR) and 90-day unfavorable outcomes after MT; however, whether NHR linked to early complications after MT remains unclear (Ci et al., 2025). Hence, clinicians or researchers may still be uncertain about the potential utility of NHR as a marker for assessing HT and MCE in this context. To address this gap, we conducted this retrospective study evaluating associations between six common inflammatory-lipid indices, including monocyte-to-HDL-C ratio (MHR), platelet-to-HDL-C ratio (PHR), NHR, lymphocyte-to-HDL-C ratio (LHR), fibrinogen-to-HDL-C ratio (FHR), and C-reactive protein-to-HDL-C ratio (CRP/HDL-C), and four common post-MT clinical outcomes, namely, HT, MCE, 90-day unfavorable outcome, and all-cause mortality. We also aimed to identify indices that demonstrated consistent associations with all four clinical outcomes.

## 2 Methods

### 2.1 Study design

This study was approved by our institutional ethics committee (KYLLKS20260127) and registered in the National Medical Research Registration and Filling System (www.medicalresearch.org.cn; registration number: MR-51-26-034299). All patients with AIS treated by MT at Suinning Central Hospital from January 2022 to December 2025 were retrospectively reviewed. The inclusion criteria were as follows: (1) acute occlusion of the internal carotid artery (ICA) or the M1/M2 segment of the middle cerebral artery (MCA); (2) time from onset or last known well to admission < 24 h; and (3) age >18 years. Patients were excluded if they had (1) artery occlusion in the posterior circulation, involving the vertebral artery, basilar artery, or posterior cerebral artery; (2) severe or progressive comorbidity at current onset, especially hepatic dysfunction; (3) immune disease or current use of immune-related drugs; (4) active malignancy; (5) previous neurological disease with obvious residual disability (modified Rankin Scale [mRS] score >1); and (6) occurrence of other neurological injury within 90 days follow-up.

### 2.2 Patient management

Patients were managed in accordance with the Chinese Stroke Association guidelines for AIS (Liu et al., 2020). All patients with suspected stroke, arriving at our stroke center, received an emergent non-contrast head computed tomography (CT) to rule out intracerebral hemorrhage, followed by CT angiography (CTA) to assess the site of vessel occlusion. Blood samples for laboratory examinations were obtained before therapy. For patients presenting within 4.5 h of stroke onset, intravenous thrombolysis (IVT) with alteplase was administered after excluding contraindications. For patients with symptom occurrence or last known well time >6 h, CT perfusion (CTP) was performed to evaluate the mismatch between ischemic penumbra and infarct core. Penumbra volume ≥15 mL and mismatch ratio ≥1.8 were considered for MT. Furthermore, direct MT was performed in patients with failure or contraindications of IVT, or within the 4.5-to-6-h time window of stroke onset. All MT procedures were performed under general anesthesia by the same interventional team, using aspiration, stent retriever, or combined approaches as appropriate. Balloon angioplasty and/or stent placement for stenosis and dissection were determined at the surgeons’ discretion. After MT, patients were transferred to the neuro-intensive care unit. Scheduled dual-energy head CT scans were obtained immediately, 24 h, and 48 h after MT. Emergent CT scan was also performed when neurological deterioration occurred.

### 2.3 Data collection

The blood parameters included C-reactive protein (CRP), complete blood count, albumin, electrolytes, coagulation indices, and lipid profiles. Smoking was defined as the daily consumption of at least one cigarette for more than 1 year, and drinking referred to the daily intake of at least 80 grams of alcohol (Sun et al., 2017; Chen et al., 2023). Medical history was obtained from previous medical records, patients or family members’s interviews, or information on current drug use. The National Institute of Health Stroke Scale (NIHSS) was used for the assessment of neurological injury. The non-contrast head CT scans at admission were independently reviewed by two neuroradiologists to evaluate the stroke burden using the standard Alberta Stroke Program Early CT Score (ASPECTS) (Pexman et al., 2001), and identify the presence of hyperdense MCA sign (HMCAS), defined as MCA attenuation > 43 Hounsfield units and >1.2 times the density of the normal contralateral vessel (Koo et al., 2000). The etiology of stroke was classified according to the Trial of ORG 10172 in Acute Stroke Treatment (TOAST) classification, and categorized as large-artery atherosclerosis (LAA), cardioembolic, and other undetermined thrombi in this study (Adams et al., 1993). Collateral circulation was assessed through digital subtraction angiography at the initial phase of the MT procedure and classified as grade 0: collateral filling less than 1/3 of the occluded territory; grade 1: filling of 1/3 to 2/3 of the occluded region; and grade 2: filling more than 2/3 of the occluded territory (Christoforidis et al., 2005). Successful recanalization was defined as modified thrombolysis in cerebral infarction (mTICI) ≥2b (Zaidat et al., 2013). The onset-to-recanalization time was measured from symptom occurrence or last known well to recanalization or, to termination of the MT procedure if recanalization was not achieved (Huang et al., 2019a).

### 2.4 Inflammatory-lipid indices and outcome measurements

The inflammatory-lipid indices included in this study were selected based on previous reports and calculated from peripheral blood parameters measured at admission, involving monocyte, platelet, neutrophil, lymphocyte, fibrinogen, CRP, and HDL-C. The formulas were as follows: MHR = monocyte count/HDL-C ratio; PHR = platelet count/HDL-C ratio; NHR = neutrophil count/HDL-C ratio; LHR = lymphocyte count/HDL-C ratio; FHR = fibrinogen/HDL-C ratio; and CRP/HDL-C ratio (Li et al., 2021; Xu et al., 2026). Clinical outcomes included HT and MCE developed within 48 h after MT, as well as 90-day unfavorable outcome and all-cause mortality. HT was diagnosed based on the European Cooperative Acute Stroke Study (ECASS) II criteria, including hemorrhagic infarction (HI1/HI2) and parenchymal hematoma (PH1/PH2) (Hacke et al., 1998). Since all MT procedures were performed under general anesthesia, and some patients received post-MT sedation, MCE was primarily diagnosed based on imaging criteria: (1) hypodensity involving ≥50% of the MCE territory, accompanied by local mass effect, such as compression of the lateral ventricle and effacement of the cerebral sulci; (2) midline shift ≥5 mm at the level of the septum pellucidum or pineal gland with obliteration of the basal cistern; and (3) swelling of more than 1/3 of the cerebral hemisphere with evident midline shift (Huang et al., 2019b; Zhang et al., 2023; Zeng et al., 2022; Wiącek et al., 2022; Huang et al., 2021). The 90-day outcomes were evaluated using the mRS by trained personnel or physicians through in-person or telephone interviews. Specifically, an unfavorable outcome was defined as an mRS score >2, and all-cause mortality was defined as mRS score = 6.

### 2.5 Statistical analysis

Variables selected in this study were based on previous reports as well as our clinical experience (Qian et al., 2024; Tang et al., 2023; Genceviciute et al., 2022). Missing data patterns were shown in Supplementary Figure S1A. Considering that the proportion of missing data for each variable was less than 5% (0–4.6%), missing values were imputed using the random forest imputation method with the “missForest” package in R software (version 4.4.2, https://www.R-project.org/). All variables and clinical outcomes were enrolled into the imputation model. The random forest parameters were set as follows: ntree = 300, mtry = 10, and a random seed of set.seed (123). Comparisons of variables with missing values before and after imputation were shown in Supplementary Figure S1B. All continuous variables were non-normally distributed, confirmed by Shapiro–Wilk test, and were presented as median [interquartile range (IQR)]. Comparisons were performed using Mann–Whitney *U* test. Categorical variables were expressed as number (percentage), and were compared using Chi-square test. The discriminatory capacity of the inflammatory-lipid indices for clinical outcomes was evaluated with receiver operating characteristic (ROC) curves and quantified using area under the curve (AUC). In the primary analysis, regression models were separately constructed for the four clinical outcomes, and covariates were sex, age, and variables that met significance in univariate analysis (*p* < 0.05). The number of covariates in each model was constrained to maintain an events-per-variable ratio >10. Specific covariates included in each model were listed in Supplementary Table S1. Internal validation of these models using the bootstrap method with 1,000 resample indicated acceptable overfitting of these models (Supplementary Table S2). Collinearity among covariates was examined using the variance inflation factor (VIF), with VIF > 5 indicating substantial collinearity. Multivariable logistic regression analysis was performed to revealassociations of inflammatory-lipid indices with HT, MCE, and unfavorable outcome, which were quantified using odds ratio (OR) and 95% confidence interval (CI). Because all the inflammatory-lipid indices satisfied the proportional hazard assumption, assessed by scale Schoenfeld residuals, the multivariable Cox proportional hazard model was used to evaluate relationships between inflammatory-lipid indices and all-cause mortality, yielding hazard ratio (HR) and 95% CI. Given that data-driven covariate selection may overlook critical confounders related to pathological mechanism such as reperfusion injury and blood–brain barrier (BBB) disruption, we performed a prespecified sensitivity analysis using clinically and biologically relevant covariates, including age, sex, the initial NIHSS score, baseline ASPECTS, and additional outcome-specific covariates, selected based on prior evidence (Supplementary Table S3) (Zhang et al., 2023; Zeng et al., 2022; Huang et al., 2019b; Qian et al., 2025b). For multiple comparisons, the Benjamini-Hochberg false discovery rate (FDR) correction was applied to reduce the risk of false positives.

When analyzed as a continuous variable, NHR was the only inflammatory-lipid index that remained significantly associated with all four clinical outcomes after FDR correction and was included for further exploratory analyses. Patients were categorized into quartiles based on the NHR values. With the lowest quartile as the reference, the associations between higher quartiles and clinical outcomes were explored. Meanwhile, the median value of each quartile was treated as a continuous variable for linear trend test. The restricted cubic spline (RCS) curves were plotted to investigate any non-linear relationship between NHR and clinical outcomes. Sensitivity analysis was performed in patients with complete data to assess the robustness of the relationships. Moreover, considering that PH2 is the most severe subtype of HT, association between NHR and PH2 was also evaluated. Based on pathophysiological and practical considerations, a planned subgroup analysis was conducted with stratification of sex (female or male), age (>70 or ≤70 years), IVT (yes or on), occluded vessel (ICA or MCA), TOAST classification (LAA or cardioembolism), onset to recanalization time (>350 or ≤350 min), and collateral scores (grade 0–2). Interactions between above stratification variables and NHR were examined using the likelihood ratio test. In addition, we added NHR to basic regression models and assessed the incremental discriminatory ability for clinical outcomes bychanges in the C-statistic, category-free net reclassification improvement (NRI), and integrated discrimination improvement (IDI). Finally, mediation analysis was performed to explore whether early complications (HT and MCE) mediated the relationships between NHR and 90-day outcomes (unfavorable outcome and all-cause mortality). All statistical analyses were conducted with R software (version 4.4.2, https://www.R-project.org/). A p-FDR value <0.05 for multiple comparisons, and a *p*-value <0.05 for other comparisons were considered statistically significant.

## 3 Results

### 3.1 Baseline characteristics

A total of 513 patients were included in this study. The selection process was shown in Figure 1. Baseline characteristics of these patients were summarized in Table 1, demonstrating that 231 (45.0%) patients were female, and the median age was 72 [IQR 63–78] years. The rates of HT and MCE were 30.6% (157) and 22.6% (116), respectively. For 90-day outcomes, 281 (54.8%) patients had unfavorable outcomes, and all-cause mortality occurred in 133 (25.9%) patients. The inflammatory-lipid indices in the whole cohort were as follows: MHR 0.34 [IQR 0.22–0.49], PHR 118.38 [IQR 86.02–163.48], NHR 5.20 [IQR 3.66–7.53], LHR 0.89 [IQR 0.60–1.32], FHR 2.20 [IQR 1.72–2.94], and CRP/HDL-C 3.14 [IQR 0.99–8.53]. However, the ROC curves showed only weak-to-moderate discriminatory capacity of inflammatory-lipid indices for clinical outcomes (AUC value: HT 0.501–0.639; MCE 0.511–0.641; unfavorable outcome 0.535–0.664; all-cause mortality 0.515–0.626) (Figure 2).

**Figure 1:**
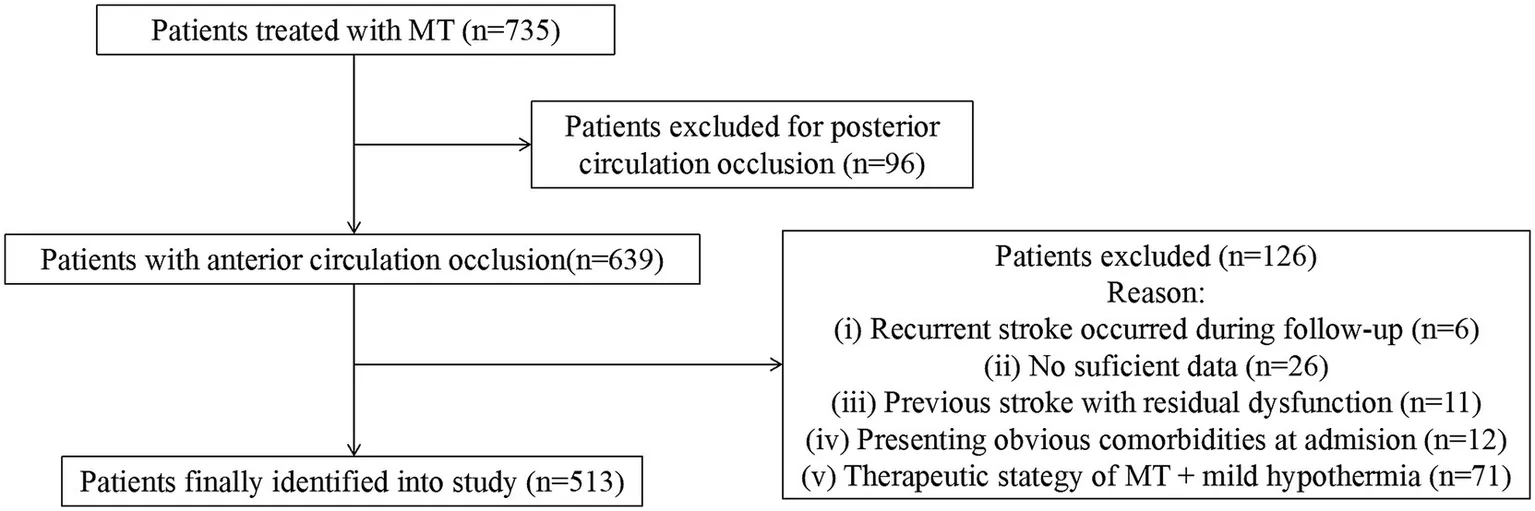
Process of patient selection.

**Table 1:** Baseline characteristics of patients with and without hemorrhagic transformation.

Variables	All	Hemorrhagic transformation	Non hemorrhagic transformation	*p*-value
Patients, *n* (%)	513	157 (30.6)	356 (69.4)	—
Demongraphics				
Female, *n* (%)	231 (45.0)	81 (51.6)	150 (42.1)	0.059
Age (years)	72 [63–78]	73 [67–79]	72 [61–78]	0.102
Smoker, *n* (%)	102 (19.9)	32 (20.4)	70 (19.7)	0.946
Alcoholism, *n* (%)	85 (16.6)	23 (14.6)	62 (17.4)	0.517
Medical history, *n* (%)				
Hypertension	249 (48.5)	77 (49.0)	172 (48.3)	0.955
Diabetes mellitus	109 (21.2)	36 (22.9)	73 (20.5)	0.616
Coronary heart disease	64 (12.5)	20 (12.7)	44 (12.4)	1.000
Atrial fibrillation	257 (50.1)	84 (53.5)	173 (48.6)	0.353
Rheumatic heart disease	42 (8.2)	17 (10.8)	25 (7.0)	0.203
Heart failure	34 (6.6)	9 (5.7)	25 (7.0)	0.727
Prior stroke	67 (13.1)	22 (14.0)	45 (12.6)	0.777
Antiplatelet at onset	24 (4.7)	9 (5.7)	15 (4.2)	0.600
Anticoagulant at onset	56 (10.9)	14 (8.9)	42 (11.8)	0.418
Clinical and imaging characteristics				
Systolic pressure (mmhg)	144 [125–162]	147 [128–164]	144 [124–160]	0.277
Diastolic pressure (mmhg)	83 [75–94]	85 [77–95]	83 [74–93]	0.075
Intravenous thrombolysis, *n* (%)	185 (36.1)	74 (47.1)	111 (31.2)	**0.001**
Initial NIHSS score	15 [12–19]	17 [13–21]	14 [10–18]	**<0.001**
Baseline ASPECTS	8 [7–9]	8 [7–9]	8 [8–9]	**<0.001**
Presence of HMCAS, *n* (%)	192 (37.4)	86 (54.8)	106 (29.8)	**<0.001**
Occluded vessel region, *n* (%)				**0.012**
ICA	183 (35.7)	69 (43.9)	114 (32.0)	
MCA	330 (64.3)	88 (56.1)	242 (68.0)	
TOAST classification, *n* (%)				0.821
LAA	187 (36.5)	55 (35.0)	132 (37.1)	
Cardioembolic	287 (55.9)	91 (58.0)	196 (55.1)	
Undetermined or others	39 (7.6)	11 (7.0)	28 (7.9)	
Collateral score, *n* (%)				**0.001**
Grade 0	136 (26.5)	59 (37.6)	77 (21.6)	
Grade 1	210 (40.9)	52 (33.1)	158 (44.4)	
Grade 2	167 (32.6)	46 (29.3)	121 (34.0)	
Onset to recanalization time (min)	354 [280–455]	386 [305–495]	341 [271–427]	**<0.001**
Stent implantation, *n* (%)	116 (22.6)	30 (19.1)	86 (24.2)	0.252
Successful recanalization, *n* (%)	471 (91.8)	136 (86.6)	335 (94.1)	**0.008**
Laboratory findings				
Total calcium (mmol/L)	2.23 [2.14–2.31]	2.23 [2.14–2.30]	2.23 [2.15–2.32]	0.968
Sodium (mmol/L)	139.00 [137.00–140.80]	138.90 [136.50–140.60]	139.00 [137.10–140.93]	0.175
Potassium (mmol/L)	3.80 [3.50–4.06]	3.80 [3.45–4.04]	3.82 [3.53–4.08]	0.237
Chloride (mmol/L)	104.50 [102.40–106.70]	104.30 [102.60–106.60]	104.50 [102.40–106.73]	0.912
Glucose (mmol/L)	7.30 [6.20–9.30]	8.30 [6.80–10.40]	7.00 [6.10–8.82]	**<0.001**
CRP (mg/L)	3.93 [1.26–10.68]	6.78 [2.38–14.62]	3.08 [1.08–8.15]	**<0.001**
White blood cell, × 10^9^/L	8.80 [7.20–10.80]	9.10 [7.40–11.50]	8.60 [7.00–10.62]	0.093
Neutrophil, × 10^9^/L	7.01 [5.18–9.11]	7.84 [5.90–10.12]	6.60 [5.03–8.62]	**<0.001**
Monocyte, × 10^9^/L	0.45 [0.32–0.60]	0.44 [0.29–0.56]	0.45 [0.32–0.62]	0.199
Lymphocyte, × 10^9^/L	1.17 [0.82–1.60]	0.94 [0.67–1.40]	1.25 [0.89–1.75]	**<0.001**
Hemoglobin, g/L	127.00 [116.00–138.00]	127.00 [114.00–138.00]	127.00 [116.00–138.25]	0.534
Platelet, × 10^9^/L	158.00 [117.00–201.00]	175.00 [124.00–213.00]	151.00 [115.00–193.00]	**0.012**
PT (s)	12.00 [11.10–12.90]	12.10 [11.30–13.00]	11.90 [11.00–12.90]	0.187
TT (s)	17.40 [16.30–18.20]	17.60 [16.40–18.40]	17.20 [16.30–18.20]	0.094
APTT (s)	27.90 [25.30–31.60]	27.90 [25.20–32.00]	27.80 [25.60–31.60]	0.909
Fibrinogen (g/L)	2.88 [2.41–3.35]	2.85 [2.39–3.16]	2.90 [2.44–3.45]	0.225
INR	1.02 [0.96–1.10]	1.04 [0.97–1.10]	1.01 [0.95–1.09]	0.166
Albumin (g/L)	39.60 [37.10–42.10]	39.80 [37.20–42.10]	39.50 [37.10–42.02]	0.880
TC (mmol/L)	4.43 [3.82–5.13]	4.50 [3.85–5.11]	4.39 [3.79–5.14]	0.883
TG (mmol/L)	1.19 [0.88–1.78]	1.20 [0.91–1.79]	1.19 [0.87–1.76]	0.400
LDL-C (mmol/L)	2.18 [1.65–2.67]	2.18 [1.64–2.61]	2.17 [1.67–2.69]	0.958
HDL-C (mmol/L)	1.31 [1.11–1.51]	1.28 [1.05–1.47]	1.33 [1.13–1.54]	**0.048**

Bold values indicate statistical significance.

NIHSS, National Institute of Health Stroke Scale; ASPECTS, Alberta Stroke Program Early CT Score; HMCAS, Hyperdense middle cerebral artery sign; ICA, Internal carotid artery; MCA, Middle cerebral artery; TOAST, Trial of ORG 10172 in Acute Stroke Treatment; LAA, Large-artery atherosclerosis; CRP, C-reactive protein; PT, Prothrombin time; TT, Thrombin time; APTT, Activated partial thromboplastin time; INR, International normalized ratio; TC, Total cholesterol; TG, Triglyceride; HDL-C, High-density lipoprotein cholesterol; LDL-C, Low-density lipoprotein cholesterol.

**Figure 2:**
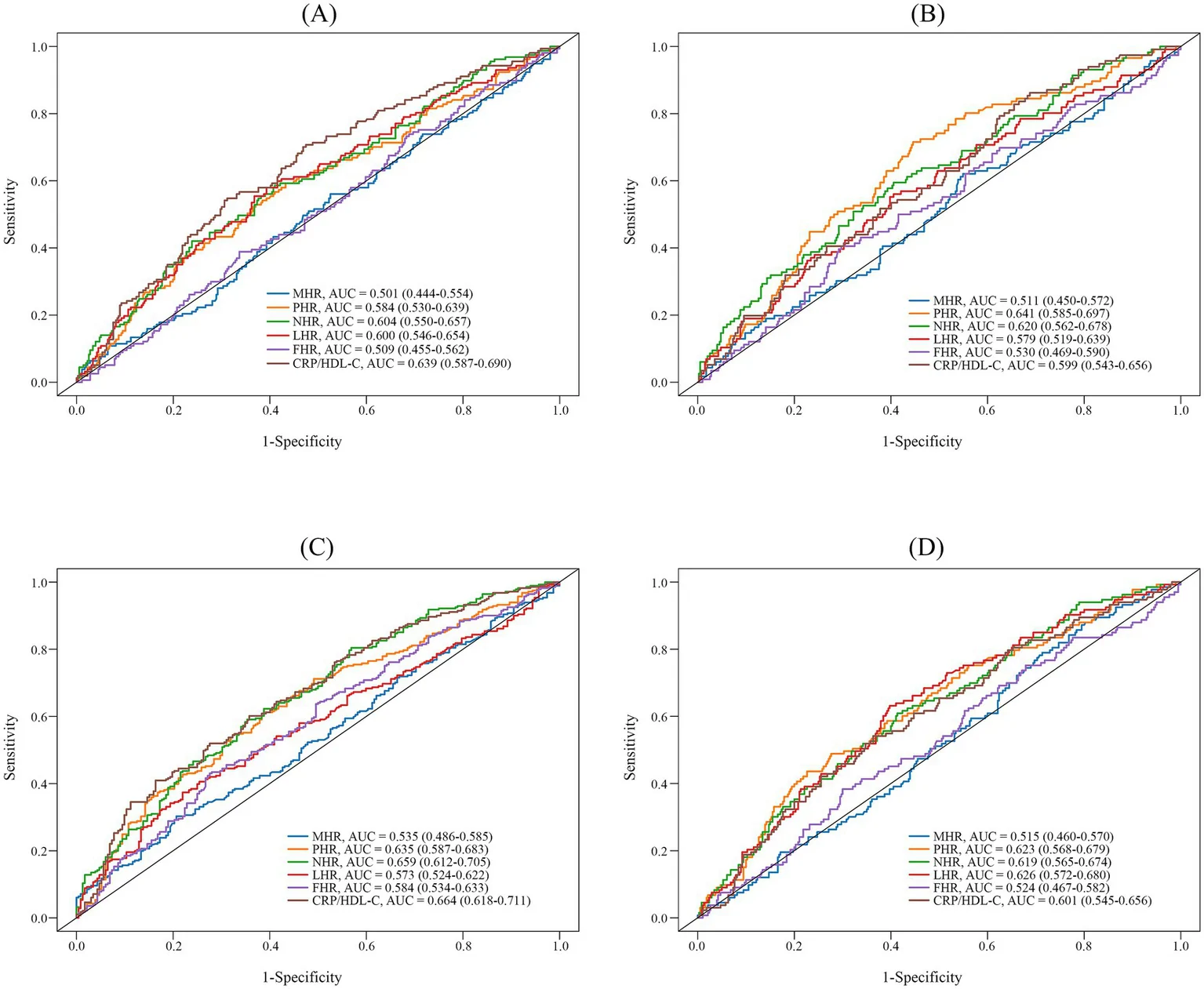
ROC curves demonstrated the discriminatory ability of inflammatory-lipid indices for clinical outcomes. **(A)** Hemorrhagic transformation; **(B)** malignant cerebral edema; **(C)** unfavorable outcome; **(D)** all-cause mortality.

### 3.2 Association between inflammatory-lipid indices and hemorrhagic transformation

Comparisons between patients with and without HT were summarized in Table 1. We found that patients with HT had higher proportions of IVT, ICA occlusion, presence of HMCAS, higher initial NIHSS score, and lower baseline ASPECTS, than those without HT. These patients also had a lower rate of successful recanalization, longer time of onset-to-recanalization, and poorer collateral circulation (all *p* < 0.05). For inflammatory-lipid indices, patients with HT had significantly higher PHR (*p* = 0.002), NHR (*p* < 0.001), and CRP/HDL-C (*p* < 0.001), and lower LHR (*p* < 0.001) (Table 2). The VIF for all covariates was < 2, suggesting limited collinearity within the models (Supplementary Figure S2). Multivariable regression analysis revealed that, after adjusting for sex, age, the initial NIHSS score, baseline ASPECTS, onset to recanalization time, intravenous thrombolysis, occluded vessel region, collateral score, presence of HMCAS, successful recanalization, and glucose, only NHR (OR 1.079, 95% CI 1.023–1.142, p-FDR = 0.034) was significantly associated with HT (Table 3).

**Table 2:** Comparisons of inflammatory-lipid indices between patients with and without adverse clinical outcomes.

Inflammatory-lipid indices	Hemorrhagic transformation			Malignant cerebral edema		
	Yes	No	*p*-value	Yes	No	*p*-value
MHR	0.34 [0.24–0.50]	0.35 [0.22–0.49]	0.983	0.34 [0.21–0.5]	0.34 [0.22–0.49]	0.712
PHR	135.38 [91.60–188.50]	113.61 [84.34–153.66]	0.002	145.58 [110.99–186.95]	112.2 [84.06–153.66]	<0.001
NHR	5.95 [4.12–8.77]	5.00 [3.55–6.89]	<0.001	6.42 [4.35–9.12]	5.03 [3.55–7.08]	<0.001
LHR	0.78 [0.53–1.19]	0.95 [0.66–1.39]	<0.001	0.79 [0.53–1.17]	0.94 [0.64–1.36]	0.009
FHR	2.21 [1.79–2.98]	2.20 [1.72–2.89]	0.745	2.33 [1.77–3.01]	2.20 [1.71–2.88]	0.325
CRP/HDL-C	5.24 [1.78–12.08]	2.43 [0.81–6.74]	<0.001	4.63 [1.6–11.02]	2.86 [0.86–7.73]	0.001

	Unfavorable outcome			All-cause mortality		
	Yes	No	*p*-value	Yes	No	*p*-value
MHR	0.36 [0.23–0.52]	0.34 [0.21–0.47]	0.161	0.36 [0.24–0.49]	0.34 [0.21–0.49]	0.588
PHR	134.18 [96.97–181.43]	103.86 [81.57–141.00]	<0.001	139.07 [105.41–191.03]	112.99 [84.34–152.57]	<0.001
NHR	5.93 [4.34–8.62]	4.60 [3.20–6.33]	<0.001	6.25 [4.36–8.88]	4.98 [3.54–7.01]	<0.001
LHR	0.82 [0.54–1.29]	0.96 [0.70–1.37]	0.004	0.76 [0.52–1.03]	0.96 [0.65–1.41]	<0.001
FHR	2.32 [1.82–3.05]	2.05 [1.57–2.65]	0.001	2.22 [1.82–3.01]	2.20 [1.71–2.88]	0.392
CRP/HDL-C	4.89 [1.58–11.59]	1.87 [0.64–5.23]	<0.001	4.93 [1.55–11.33]	2.69 [0.86–7.51]	<0.001

MHR, monocyte-to-HDL-C ratio; PHR, platelet-to-HDL-C ratio; NHR, neutrophil-to-HDL-C ratio; LHR, lymphocyte-to-HDL-C ratio; FHR, fibrinogen-to-HDL-C ratio; CRP/HDL-C, C-reactive protein-to-HDL-C ratio; HDL-C, High-density lipoprotein cholesterol.

**Table 3:** Multivariable analyses revealing the associations between the inflammatory-lipid indices and clinical outcomes.

Inflammatory-lipid indices	Hemorrhagic transformation^a^			Malignant cerebral edema^b^			Unfavorable outcome^c^			All-cause mortality^d^		
	OR (95% CI)	*p*-value	p-FDR value	OR (95% CI)	*p*-value	p-FDR value	OR (95% CI)	*p*-value	p-FDR value	HR (95% CI)	*p*-value	p-FDR value
Crude												
MHR	1.069 (0.499–2.209)	0.857	0.857	1.051 (0.449–2.304)	0.903	0.903	2.539 (1.220–5.562)	0.015	0.019	1.279 (0.664–2.461)	0.460	0.552
PHR*	1.463 (1.132–1.906)	0.003	0.011	1.792 (1.361–2.39)	<0.001	<0.001	1.894 (1.424–2.570)	<0.001	<0.001	1.467 (1.231–1.748)	<0.001	<0.001
NHR	1.099 (1.046–1.158)	<0.001	0.001	1.115 (1.058–1.177)	<0.001	<0.001	1.205 (1.132–1.289)	<0.001	<0.001	1.064 (1.032–1.096)	<0.001	<0.001
LHR	0.632 (0.450–0.863)	0.005	0.011	0.720 (0.500–1.004)	0.064	0.097	0.792 (0.608–1.026)	0.079	0.079	0.535 (0.381–0.750)	<0.001	<0.001
FHR	0.976 (0.830–1.137)	0.768	0.857	1.033 (0.869–1.215)	0.694	0.833	1.269 (1.087–1.498)	0.003	0.005	1.018 (0.890–1.165)	0.787	0.787
CRP/HDL-C	1.016 (1.004–1.030)	0.009	0.013	1.012 (1.001–1.025)	0.039	0.079	1.030 (1.013–1.050)	0.001	0.002	1.005 (0.996–1.014)	0.247	0.371
Adjusted												
MHR	0.855 (0.364–1.985)	0.717	0.717	0.775 (0.311–1.817)	0.569	0.683	2.663 (1.099–6.796)	0.034	0.041	0.879 (0.433–1.785)	0.723	0.723
PHR*	1.294 (0.963–1.738)	0.084	0.127	1.690 (1.247–2.298)	<0.001	0.004	1.646 (1.205–2.282)	0.002	0.006	1.340 (1.094–1.641)	0.004	0.022
NHR	1.079 (1.023–1.142)	0.005	0.034	1.089 (1.031–1.154)	0.002	0.008	1.177 (1.098–1.269)	<0.001	<0.001	1.047 (1.012–1.084)	0.007	0.022
LHR	0.675 (0.474–0.942)	0.024	0.073	0.817 (0.564–1.149)	0.263	0.395	1.015 (0.738–1.396)	0.923	0.923	0.680 (0.486–0.950)	0.024	0.048
FHR	0.961 (0.799–1.142)	0.667	0.717	0.994 (0.823–1.184)	0.952	0.952	1.266 (1.058–1.532)	0.012	0.018	0.959 (0.833–1.105)	0.568	0.723
CRP/HDL-C	1.013 (0.999–1.028)	0.056	0.113	1.015 (1.001–1.028)	0.028	0.056	1.023 (1.006–1.044)	0.012	0.018	1.002 (0.992–1.011)	0.671	0.723

^*^Per 100 units.

a Adjusted for sex, age, initial NIHSS score, baseline ASPECTS, onset to recanalization time, intravenous thrombolysis, occluded vessel region, collateral score, presence of HMCAS, successful recanalization, and glucose.

b Adjusted for sex, age, initial NIHSS score, baseline ASPECTS, history of hypertension, occluded vessel region, collateral score, presence of HMCAS, sodium, and glucose.

c Adjusted for sex, age, initial NIHSS score, baseline ASPECTS, onset to recanalization time, history of hypertension, collateral score, presence of HMCAS, successful recanalization, total calcium, sodium, glucose, and TG.

d Adjusted for sex, age, smoker, initial NIHSS score, baseline ASPECTS, occluded vessel region, systolic pressure, collateral score, presence of HMCAS, successful recanalization, sodium, and glucose.

MHR, monocyte-to-HDL-C ratio; PHR, platelet-to-HDL-C ratio; NHR, neutrophil-to-HDL-C ratio; LHR, lymphocyte-to-HDL-C ratio; FHR, fibrinogen-to-HDL-C ratio; CRP/HDL-C, C-reactive protein-to-HDL-C ratio; HDL-C, High-density lipoprotein cholesterol; OR, Odds ratio; HR, Hazard ratio; CI, Confidence interval; NIHSS, National Institute of Health Stroke Scale; ASPECTS, Alberta Stroke Program Early CT Score; HMCAS, Hyperdense middle cerebral artery sign; TG, Triglyceride.

### 3.3 Association between inflammatory-lipid indices and malignant cerebral infarction

Baseline characteristics of patients with and without MCE were listed in Supplementary Table S4. Compared with patients without MCE, those with MCE were older, had higher proportion of female, history of hypertension, and presence of HMCAS, higher initial NIHSS score, lower baseline ASPECTS, and worse collateral status (all *p* < 0.05). Critically, these patients had higher levels of PHR (*p* < 0.001), NHR (*p* < 0.001), and CRP/HDL-C (*p* = 0.001), as well as a lower level of LHR (*p* = 0.009) (Table 2). After adjusting for sex, age, the initial NIHSS score, baseline ASPECTS, history of hypertension, occluded vessel region, collateral score, presence of HMCAS, sodium, and glucose, multivariable regression analysis indicated that PHR (per 100 units, OR 1.690, 95% CI 1.247–2.298, p-FDR = 0.004) and NHR (OR 1.089, 95% CI 1.031–1.154, p-FDR = 0.008) were significantly associated with MCE (Table 3).

### 3.4 Association between inflammatory-lipid indices and 90-day unfavorable outcome

Patients with 90-day unfavorable outcome were older, had higher initial NIHSS score, lower baseline ASPECTS, worse collateral circulation, and longer time of onset to recanalization, compared to those with favorable outcome. Moreover, these patients had higher proportions of history of hypertension, and presence of HMCAS, as well as a lower rate of successful recanalization (all *p* < 0.05) (Supplementary Table S5). Furthermore, patients with unfavorable outcome exhibited significantly elevated levels of PHR (*p* < 0.001), NHR (*p* < 0.001), FHR (*p* = 0.001), and CRP/HDL-C (*p* < 0.001), and reduced LHR (*p* = 0.004), compared with those with favorable outcome. After adjusting for sex, age, the initial NIHSS score, baseline ASPECTS, onset to recanalization time, history of hypertension, collateral score, presence of HMCAS, successful recanalization, total calcium, sodium, glucose, and triglyceride, multivariable analysis indicated that MHR (OR 2.663, 95% CI 1.099–6.796, p-FDR = 0.041), PHR (per 100 units, OR 1.646, 95% CI 1.205–2.282, p-FDR = 0.006), NHR (OR 1.177, 95% CI 1.098–1.269, p-FDR < 0.001), FHR (OR 1.266, 95% CI 1.058–1.532, p-FDR = 0.018), and CRP/HDL-C (OR 1.023, 95% CI 1.006–1.044, p-FDR = 0.018) remained significantly linked to 90-day unfavorable outcome (Table 3).

### 3.5 Association between inflammatory-lipid indices and 90-day all-cause mortality

Baseline characteristics of 90-day survivors and non-survivors were summarized in Supplementary Table S6. Univariate Cox analysis showed that sex, age, smoker, systolic pressure, the initial NIHSS score, baseline ASPECTS, presence of HMCAS, occluded vessel, collateral circulation, and successful recanalization were significantly associated with 90-day all-cause mortality (all *p* < 0.05). Compared with survivors, non-survivors had higher PHR (*p* < 0.001), NHR (*p* < 0.001), and CRP/HDL-C (*p* < 0.001), and lower LHR (*p* < 0.001) (Table 2). Multivariable Cox proportional hazard regression, with adjustment for sex, age, smoker, the initial NIHSS score, baseline ASPECTS, occluded vessel region, systolic pressure, collateral score, presence of HMCAS, successful recanalization, sodium, and glucose, demonstrated that PHR (per 100 units, HR 1.340, 95% CI 1.094–1.641, p-FDR = 0.022), NHR (HR 1.047, 95% CI 1.012–1.084, p-FDR = 0.022), and LHR (HR 0.680, 95% CI 0.486–0.950, p-FDR = 0.048) were significantly associated with all-cause mortality (Table 3).

### 3.6 Sensitivity analysis adjusting for prespecified clinically and biologically relevant covariates

In the prespecified sensitivity analysis using clinically and biologically relevant covariates, we found significant associations of NHR (OR 1.079, 95% CI 1.023–1.143, p-FDR = 0.038) with HT; PHR (per 100 units, OR 1.710, 95% CI 1.263–2.324, p-FDR = 0.003) and NHR (OR 1.089, 95% CI 1.031–1.154, p-FDR = 0.008) with MCE; PHR (per 100 units, OR 1.738, 95% CI 1.275–2.413, p-FDR = 0.001), NHR (OR 1.172, 95% CI 1.095–1.261, p-FDR < 0.001), FHR (OR 1.294, 95% CI 1.082–1.564, p-FDR = 0.011), and CRP/HDL-C (OR 1.023, 95% CI 1.006–1.044, p-FDR = 0.017) with unfavorable outcome; and PHR (per 100 units, HR 1.373, 95% CI 1.122–1.680, p-FDR = 0.012), NHR (HR 1.047, 95% CI 1.012–1.082, p-FDR = 0.014) and LHR (HR 0.625, 95% CI 0.445–0.880, p-FDR = 0.014) with all-cause mortality (Supplementary Table S7).

### 3.7 Further exploratory analyses for evaluating relationships between NHR and clinical outcomes

Based on the above findings, NHR was the only inflammatory-lipid index that was found to be significantly associated with all four clinical outcomes after FDR correction, and included for further analyses. When treated as a categorical variable, using the lowest quartile as the reference, the highest quartile showed significant associations with the elevated risk of these clinical outcomes: HT: OR 2.230, 95% CI 1.219–4.137, *p* = 0.009; MCE: OR 1.979, 95% CI 1.036–3.862, *p* = 0.040; unfavorable outcome: OR 3.091, 95% CI 1.692–5.725, *p* < 0.001; all-cause mortality: HR 1.973, 95% CI 1.153–3.379, *p* = 0.013 (Table 4). The RCS curves revealed linear relationships of NHR with HT (p-non-linear = 0.840), MCE (p-non-linear = 0.749), unfavorable outcome (p-non-linear = 0.117), and all-cause mortality (p-non-linear = 0.375) (Figure 3).

**Table 4:** Associations between quartiles of NHR and clinical outcomes.

Inflammatory-lipid indices	Hemorrhagic transformation^a^		Malignant cerebral edema^b^		Unfavorable outcome^c^		All-cause mortality^d^	
	OR (95% CI)	*p* value	OR (95% CI)	*p*-value	OR (95% CI)	*p*-value	HR (95% CI)	*p*-value
Crude								
Quartile 1	Reference		Reference		Reference		Reference	
Quartile 2	1.397 (0.792–2.483)	0.249	1.182 (0.613–2.297)	0.616	1.957 (1.190–3.244)	0.008	1.493 (0.845–2.640)	0.167
Quartile 3	1.436 (0.817–2.546)	0.210	1.708 (0.920–3.231)	0.093	2.910 (1.762–4.861)	<0.001	1.809 (1.047–3.125)	0.033
Quartile 4	2.777 (1.621–4.841)	<0.001	2.637 (1.458–4.898)	0.001	3.921 (2.348–6.648)	<0.001	2.719 (1.614–4.583)	<0.001
*p* for trend		<0.001		<0.001		<0.001		<0.001
Adjusted								
Quartile 1	Reference		Reference		Reference		Reference	
Quartile 2	1.148 (0.612–2.164)	0.665	1.049 (0.517–2.136)	0.893	1.927 (1.067–3.514)	0.030	1.351 (0.746–2.447)	0.319
Quartile 3	0.979 (0.521–1.843)	0.948	1.410 (0.722–2.794)	0.317	2.795 (1.556–5.085)	<0.001	1.404 (0.795–2.479)	0.241
Quartile 4	2.230 (1.219–4.137)	0.009	1.979 (1.036–3.862)	0.040	3.091 (1.692–5.725)	<0.001	1.973 (1.153–3.379)	0.013
*p* for trend		0.005		0.017		<0.001		0.009

a Adjusted for sex, age, initial NIHSS score, baseline ASPECTS, onset to recanalization time, intravenous thrombolysis, occluded vessel region, collateral score, presence of HMCAS, successful recanalization, and glucose.

b Adjusted for sex, age, initial NIHSS score, baseline ASPECTS, history of hypertension, occluded vessel region, collateral score, presence of HMCAS, sodium, and glucose.

c Adjusted for sex, age, initial NIHSS score, baseline ASPECTS, onset to recanalization time, history of hypertension, collateral score, presence of HMCAS, successful recanalization, total calcium, sodium, glucose, and TG.

d Adjusted for sex, age, smoker, initial NIHSS score, baseline ASPECTS, occluded vessel region, systolic pressure, collateral score, presence of HMCAS, successful recanalization, sodium, and glucose.

OR, Odds ratio; HR, Hazard ratio; CI, Confidence interval; NIHSS, National Institute of Health Stroke Scale; ASPECTS, Alberta Stroke Program Early CT Score; HMCAS, Hyperdense middle cerebral artery sign; TG, Triglyceride.

**Figure 3:**
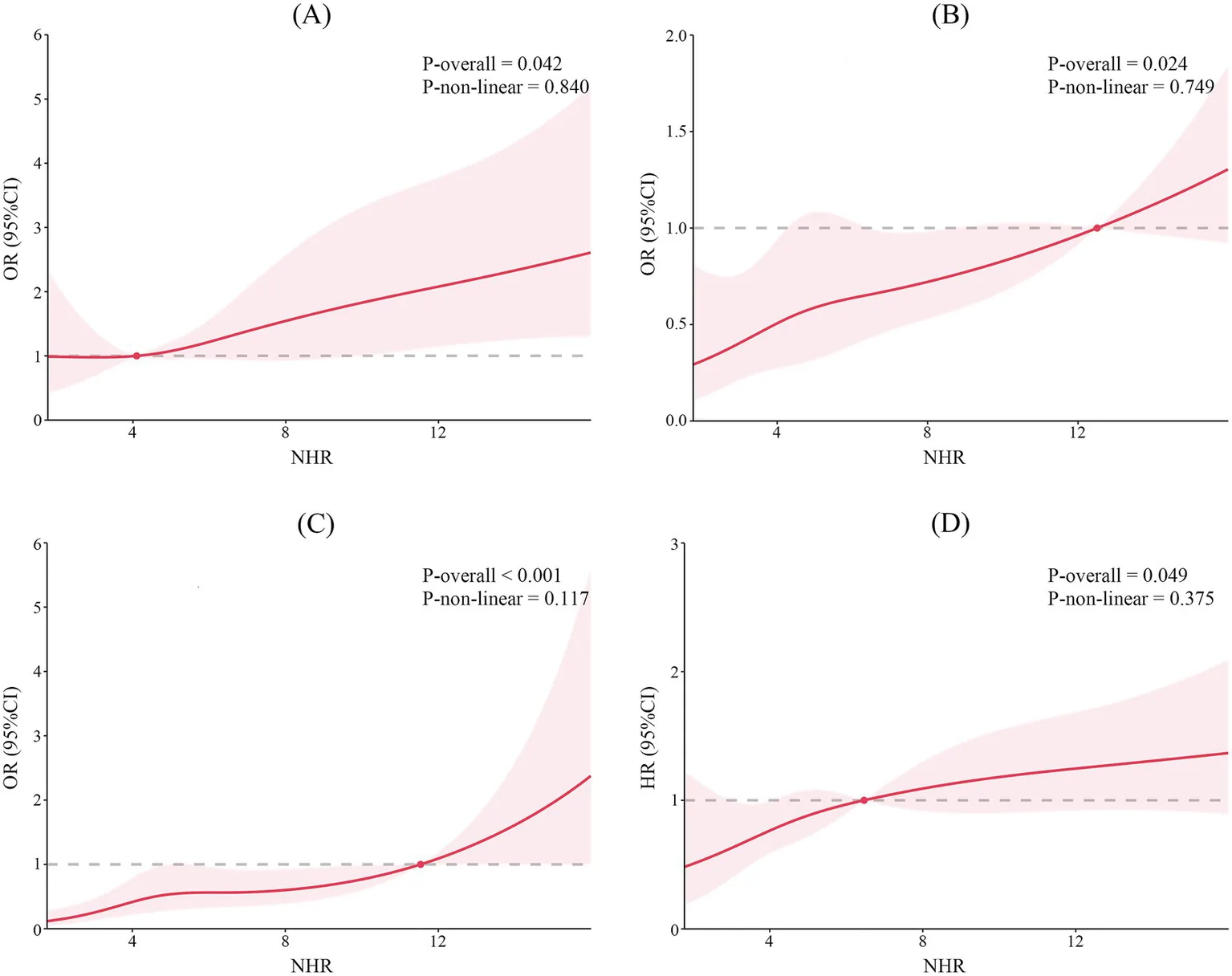
Restricted cubic spline curves for the associations between NHR and clinical outcomes. **(A)** Hemorrhagic transformation; **(B)** malignant cerebral edema; **(C)** unfavorable outcome; **(D)** all-cause mortality.

In the sensitivity analysis, a total of 488 patients had complete data. The incidences of HT, MCE, unfavorable outcome, and all-cause mortality were 30.5% (149), 23.6% (115), 55.7% (272), and 26.8% (131), respectively. Multivariable analysis also confirmed significant associations between NHR and clinical outcomes (HT: OR 1.087, 95% CI 1.029–1.152, *p* = 0.003; MCE: OR 1.086, 95% CI 1.028–1.151, *p* = 0.004; unfavorable outcome: OR 1.184, 95% CI 1.102–1.279, *p* < 0.001; all-cause mortality: HR 1.043, 95% CI 1.008–1.080, *p* = 0.015) (Table 5). In addition, PH2 occurred in 51 patients (9.9%). When adjusting for the covariates in the HT model, NHR was also significantly associated with PH2 (OR 1.107, 95% CI 1.038–1.183, *p* = 0.002).

**Table 5:** Sensitivity analyses revealing the associations between NHR and clinical outcomes in patients with complete data.

NHR	Hemorrhagic transformation^a^		Malignant cerebral edema^b^		Unfavorable outcome^c^		All-cause mortality^d^	
	OR (95% CI)	*p*-value	OR (95% CI)	*p*-value	OR (95% CI)	*p*-value	HR (95% CI)	*p*-value
Crude	1.108 (1.053–1.169)	<0.001	1.112 (1.055–1.175)	<0.001	1.211 (1.135–1.299)	<0.001	1.061 (1.029–1.094)	<0.001
Adjusted	1.087 (1.029–1.152)	0.003	1.086 (1.028–1.151)	0.004	1.184 (1.102–1.279)	<0.001	1.043 (1.008–1.080)	0.015

a Adjusted for sex, age, initial NIHSS score, baseline ASPECTS, onset to recanalization time, intravenous thrombolysis, occluded vessel region, collateral score, presence of HMCAS, successful recanalization, and glucose.

b Adjusted for sex, age, initial NIHSS score, baseline ASPECTS, history of hypertension, occluded vessel region, collateral score, presence of HMCAS, sodium, and glucose.

c Adjusted for sex, age, initial NIHSS score, baseline ASPECTS, onset to recanalization time, history of hypertension, collateral score, presence of HMCAS, successful recanalization, total calcium, sodium, glucose, and TG.

d Adjusted for sex, age, smoker, initial NIHSS score, baseline ASPECTS, occluded vessel region, systolic pressure, collateral score, presence of HMCAS, successful recanalization, sodium, and glucose.

OR, Odds ratio; HR, Hazard ratio; CI, Confidence interval; NIHSS, National Institute of Health Stroke Scale; ASPECTS, Alberta Stroke Program Early CT Score; HMCAS, Hyperdense middle cerebral artery sign; TG, Triglyceride.

In the subgroup analysis, we found a robust relationship between NHR and HT in female, and in patients aged >70 years, no IVT therapy, ICA occlusion, cardioembolic stroke, onset-to-recanalization time >350 min, and a collateral score of grade 2 (Figure 4A). Significant associations with MCE were observed in male patients and those aged >70 years, IVT therapy, MCA occlusion, cardioembolic stroke, onset-to-recanalization time ≤350 min, and a collateral score of grade 2 (Figure 4B). For unfavorable outcome, significant correlations were found in most subgroups, except for patients with a collateral score of grade 0 (Figure 4C). Regarding all-cause mortality, significant relationships with NHR were found in females, and in patients aged ≤70 years, IVT therapy, MCA occlusion, and a collateral score of grade 2 (Figure 4D). Critically, a significant interaction was observed between NHR and TOAST classification (*p* for interaction = 0.023), demonstrating that NHR was significantly associated with all-cause mortality in patients with cardioembolic stroke (HR 1.109, 95% CI 1.055–1.165, *p* < 0.001). However, after FDR correction, this interaction was no longer statistically significant (p-FDR for interaction = 0.161, Supplementary Table S8).

**Figure 4:**
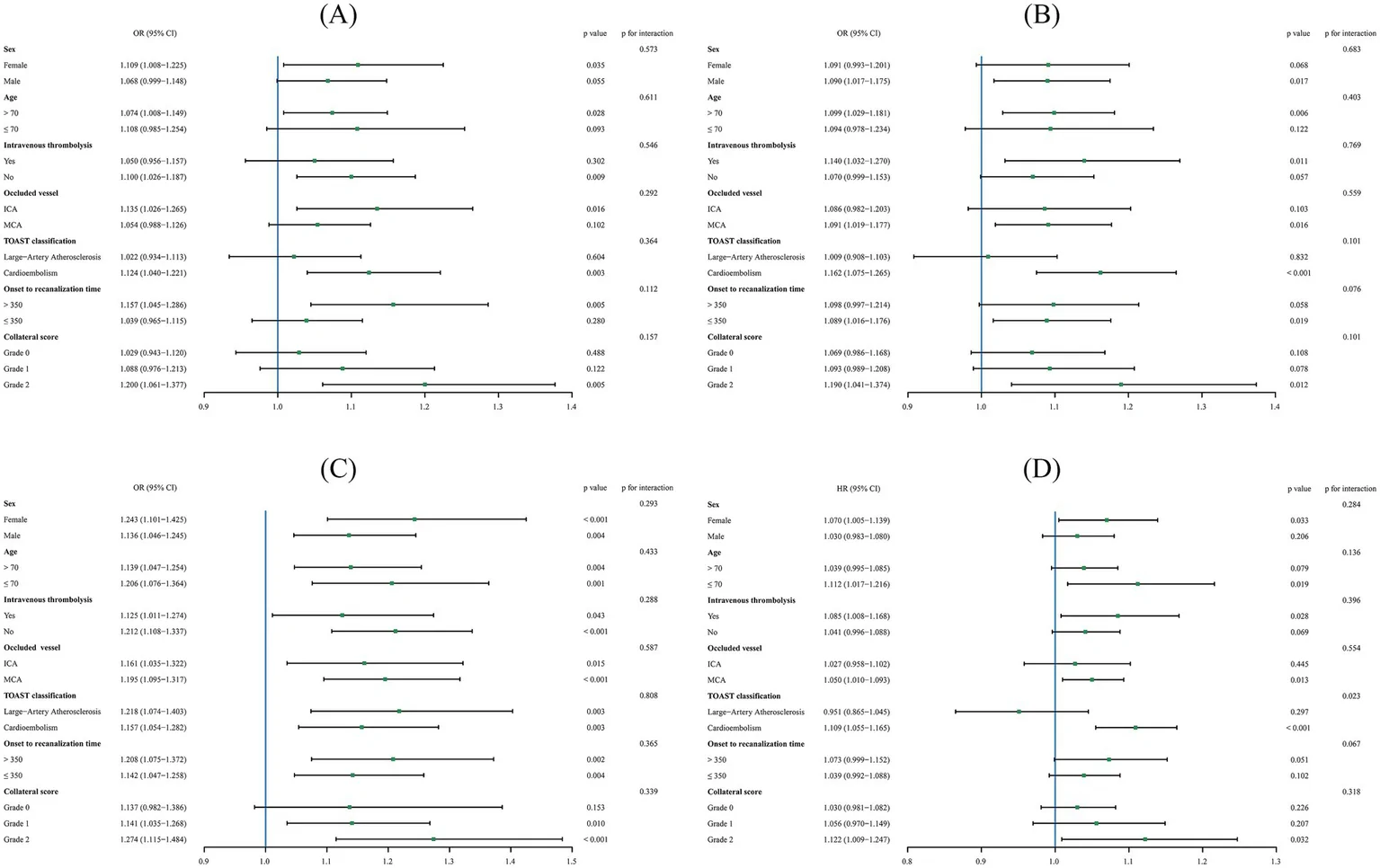
Subgroup analysis revealing the associations between NHR and clinical outcomes under different conditions. **(A)** Hemorrhagic transformation; **(B)** malignant cerebral edema; **(C)** unfavorable outcome; **(D)** all-cause mortality.

Considering the limited discriminatory ability of NHR for clinical outcomes, as shown by the ROC curves, we incorporated NHR into the regression models to evaluate the incremental discriminatory value for clinical outcomes (Table 6). For HT and MCE, we found that adding NHR to the models significantly increased category-free NRI (HT: *p* = 0.007; MCE: *p* = 0.010) and IDI (HT: *p* = 0.013; MCE: *p* = 0.009), whereas the improvement in C-statistic was modest (HT: *p* = 0.169; MCE: *p* = 0.271). For unfavorable outcome, incorporating NHR into the model significantly improved discriminatory performance, reflected by increased C-statistic (*p* = 0.043), category-free NRI (*p* < 0.001), and IDI (*p* < 0.001). Adding NHR to the model only significantly improved the category-free NRI (*p* = 0.016) for all-cause mortality; however, the increments in C-statistic (*p* = 0.394) and IDI (*p* = 0.121) were limited.

**Table 6:** Improvements of reclassification and discrimination statistics for clinical outcomes by incorporating NHR.

Models	C-statistic		Category-free NRI		IDI	
	Estimate (95% CI)	*p*-value	Estimate (95% CI), %	*p*-value	Estimate (95% CI), %	*p*-value
Hemorrhagic transformation						
Basic model^a^	0.759 (0.715–0.804)	Reference	Reference		Reference	
Basic model^a^ + NHR	0.769 (0.725–0.813)	0.169	25.0 (6.5–43.4)	0.007	1.4 (0.3–2.6)	0.013
Malignant cerebral edema						
Basic model^b^	0.740 (0.688–0.792)	Reference	Reference		Reference	
Basic model^b^ + NHR	0.750 (0.698–0.801)	0.271	26.6 (6.2–46.9)	0.010	2.1 (0.5–3.7)	0.009
Unfavorable outcome						
Basic model^c^	0.799 (0.761–0.837)	Reference	Reference		Reference	
Basic model^c^ + NHR	0.816 (0.780–0.852)	0.043	37.1 (20.5–53.7)	<0.001	3.5 (1.8–5.1)	<0.001
All-cause mortality						
Basic model^d^	0.810 (0.766–0.853)	Reference				
Basic model^d^ + NHR	0.813 (0.770–0.856)	0.394	23.8 (4.4–43.2)	0.016	0.6 (−0.1–1.5)	0.121

a Adjusted for sex, age, initial NIHSS score, baseline ASPECTS, onset to recanalization time, intravenous thrombolysis, occluded vessel region, collateral score, presence of HMCAS, successful recanalization, and glucose.

b Adjusted for sex, age, initial NIHSS score, baseline ASPECTS, history of hypertension, occluded vessel region, collateral score, presence of HMCAS, sodium, and glucose.

c Adjusted for sex, age, initial NIHSS score, baseline ASPECTS, onset to recanalization time, history of hypertension, collateral score, presence of HMCAS, successful recanalization, total calcium, sodium, glucose, and TG.

d Adjusted for sex, age, smoker, initial NIHSS score, baseline ASPECTS, occluded vessel region, systolic pressure, collateral score, presence of HMCAS, successful recanalization, sodium, and glucose.

NHR, neutrophil-to-HDL-C ratio; HDL-C, High-density lipoprotein cholesterol; OR, Odds ratio; HR, Hazard ratio; CI, Confidence interval; NIHSS, National Institute of Health Stroke Scale; ASPECTS, Alberta Stroke Program Early CT Score; HMCAS, Hyperdense middle cerebral artery sign; TG, Triglyceride; NRI, net reclassification improvement; IDI, integrated discrimination improvement.

Combining with the above findings, we further investigated whether early complications mediated the relationship between NHR at admission and 90-day outcomes. We found that HT and MCE statistically accounted for part of the associations between NHR and unfavorable outcome, with mediation proportions of 23.9 and 23.8%, respectively (Figure 5).

**Figure 5:**
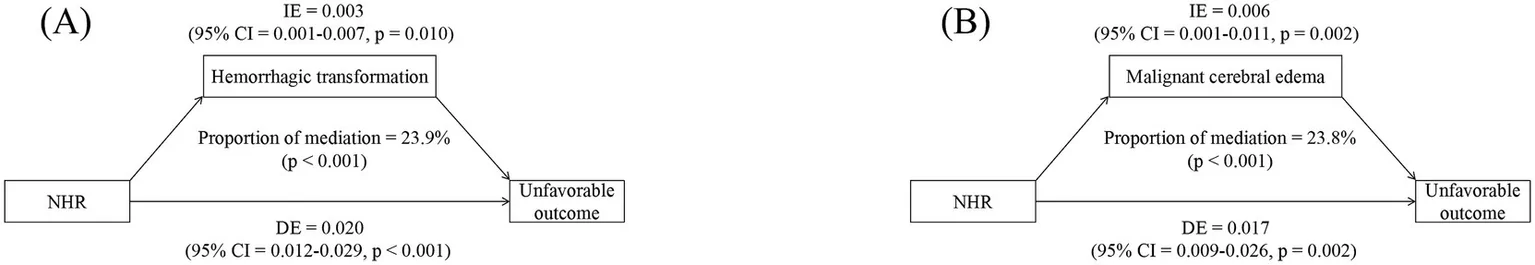
Mediation analysis indicating that hemorrhagic transformation and malignant cerebral edema statistically accounted for part of the association between NHR and 90-day unfavorable outcome; **(A)** Hemorrhagic transformation mediating NHR to unfavorable outcome; **(B)** malignant cerebral edema mediating NHR to unfavorable outcome.

## 4 Discussion

In this study, we comprehensively evaluated the associations between six common inflammatory-lipid indices and four clinical outcomes after MT. Irrespective of FDR correction, NHR was the only inflammatory-lipid index that showed a consistent significant association with all four clinical outcomes. However, the AUC values of all six indices for clinical outcomes were modest, indicating limited standalone discriminatory ability. Therefore, our study first proposed that NHR may serve as a consistent associated inflammatory-lipid marker for early HT and MCE, as well as long-term outcome following MT.

A substantial body of evidence has established the relationship between inflammatory-lipid indices, calculated from peripheral blood parameters, and post-MT outcomes (Li et al., 2021; Xu et al., 2026; Liu et al., 2026; Ci et al., 2025; Wei et al., 2025). Among them, NHR has also been extensively explored. Ci et al. reported that NHR was significantly associated with 90-day unfavorable outcome (Ci et al., 2025). However, their study did not evaluate the relationships with early post-MT complications. Wei et al. identified NHR as an independent risk factor for decompressive craniectomy after MT, but the relationship between NHR and long-term functional outcome was not explored in their study (Wei et al., 2025). Moreover, Xu et al. suggested that elevated NHR was associated with 90-day adverse functional outcome after MT, but failed to reveal the association with early complications (Xu et al., 2026). Our results corroborated and extended prior findings by demonstrating that elevated NHR was associated with an increased risk of both early post-MT complications (HT and MCE) and adverse long-term outcomes. These findings provided a comprehensive assessment of the relationship between inflammatory-lipid indices and post-MT outcomes, and clarified the NHR as a consistent indicator for outcomes in patients undergoing MT.

Inflammatory-lipid indices, which were also included in this study, have been extensively reported to be associated with specific post-MT outcomes(Li et al., 2021; Xu et al., 2026; Liu et al., 2026; Ci et al., 2025; Wei et al., 2025). However, most previous studies have focused on a single index or clinical endpoint. To address this gap, we investigated the associations between six inflammatory-lipid indices and four clinical outcomes. Through this systematic approach, NHR emerged as the index with the most consistent associations across all four outcomes. The principal contribution of this study was notmerely to re-affirm the associations of these indices or challenge their established relevanceas potential indicators, but to identify the most reliable inflammatory-lipid index for evaluating common clinical outcomes following MT in a real-world setting. The selection of NHR as a consistent inflammatory-lipid index is mainly attributable to the components, neutrophils and HDL-C, integrating dysregulated neuroinflammation and lipid metabolism, both of which are essential factors in the development and progression of AIS. Neutrophil is the earliest leukocyte subset migrating to the ischemic brain tissue after AIS, and can aggravate cellular injury and extracellular damage by releasing reactive oxygen species, proteases, and pro-inflammatory cytokines (Li et al., 2022; Zhang et al., 2022). Matrix metalloproteinase-9 released by neutrophil exacerbates the disruption of the BBB, which has been demonstrated to be a fundamental mechanism of HT and MCE in AIS (Huang et al., 2019a). In contrast, HDL-C exerts anti-atherosclerotic and anti-inflammatory effects and plays an important role in protecting the BBB after AIS (Wang et al., 2020). In addition to its role in reverse cholesterol transport, HDL-C interacts with platelets, coagulation cascade, and vascular endothelium to reduce platelet hyper-reactivity and attenuate thrombosis (Ci et al., 2025; van der Stoep et al., 2014). However, activated neutrophils can mediate the oxidation of HDL through myeloperoxidase, nicotinamide adenine dinucleotide phosphate oxidase, and nitric oxide synthase, impairing cholesterol efflux capacity and exacerbating atherogenesis and inflammation (Smith et al., 2014; Smith et al., 2017). NHR integrates neuroinflammation and dyslipidemia, and may reflect the inflammatory status and lipid metabolism of AIS patients treated with MT, potentially providing a pathophysiological basis for NHR as a risk factor for clinical outcomes in this population.

In this study, we found that NHR was associated with common early complications and late unfavorable outcomes after MT, and the linear relationships were also confirmed by RCS curves. Interestingly, in exploratory subgroup analysis for all-cause mortality, we observed a significant interaction between NHR and TOAST classification, indicating a stronger association between NHR and all-cause mortality in patients with cardioembolic stroke. However, this interaction should be interpreted with caution because multiple interaction analyses were performed. In a sensitivity analysis with FDR correction, the interaction did not remain statistically significant. Therefore, this finding should be regarded as exploratory and hypothesis-generating. Biologically, this observation may be attributed to the distinct underlying mechanisms of LAA and cardioembolism in acute LVO. We speculated that, in the process of cardioembolism, with abrupt occlusion of cerebral arteries by cardiac thrombus, in addition to the inflammatory response mediated by neutrophils, the protective capacity of HDL-C is relatively insufficient, resulting in the disruption of pro-inflammatory and anti-inflammatory balance, which may contribute to the occurrence of mortality. On the contrary, in the LAA subtype, the process vascular stenosis may enhance the cerebral ischemic tolerance, which could partially mitigate the inflammatory dysfunction reflected by NHR, accounting for the lack of significance in this subgroup. However, another possible explanation may be the limited sample size in the LAA subgroup, leading to inadequate statistical power to detect the association. Nevertheless, these findings warrant further investigation of NHR in patients with cardioembolic stroke, rather than immediate clinical application, and conclusions drawn from the subgroup with a small number of cases should be interpreted with caution.

In AIS patients treated with MT, these indices may only partially capture the inflammatory and lipid-metabolic dimensions of multifactorial pathophysiology, contributing to the modest AUC values of inflammatory-lipid indices for clinical outcomes, although significant associations with clinical outcomes were found. Therefore, using NHR as a standalone prognostic tool may be inappropriate due to its limited discriminatory ability. Thus, we incorporated NHR into regression models to assess whether it provides incremental value beyond conventional relevant factors. We found that adding NHR to the regression models increased the discriminatory capacity for unfavorable outcome, as reflected by a significant improvement in the C-statistic, category-free NRI, and IDI. The risk stratification for HT and MCE was also improved, whereas the improvement in model discrimination for all-cause mortality was limited. These results indicated that NHR should be regarded as a complementary factor within a multifactorial risk assessment framework, rather than an independent prognostic marker. The incremental value of NHR may lie in refining risk classification to more accurately identify patients at high risk.

Recognizing that early NHR is easily disturbed, the direct application of NHR in the assessment of long-term outcomes may be inappropriate. Therefore, we performed mediation analyses, which revealed that early post-MT HT and MCE statistically accounted for part of the association with 90-day unfavorable outcome, whereas the mediating effect on all-cause mortality was not significant. We believe that functional recovery is closely linked to the severity of early brain injury, which is substantially influenced by HT and MCE (Qian et al., 2025a). However, mortality, as the most extreme endpoint of adverse outcomes, is affected by more complex and broader systemic factors, which may dilute the mediating roles of early HT and MCE (Qian et al., 2024). Nevertheless, the mediation analysis in this study should be regarded as hypothesis-generating. In an observational study, mediation analysis can only reveal statistical pathways, rather than determine pathological or causal mediation. Further studies are required to elucidate the mechanisms underlying the association between NHR and all-cause mortality.

In this study, the biological and statistical dependence among the six inflammatory-lipid indices, which shared HDL-C as a common component, should be considered. HDL-C can suppress myelopoiesis and the circulating levels of monocytes, neutrophils, and platelets, while activated leukocytes can impair HDL function through oxidative modification (Westerterp et al., 2014; Bergt et al., 2001). Therefore, the cellular components are mutually regulated with HDL-C within the same inflammatory-metabolic axis, suggesting that these inflammatory-lipid indices are inherently correlated (Grao-Cruces et al., 2022; Navab et al., 2011). Because of this dependence, the relationships of the six indices with clinical outcomes should be interpreted as identifying the index with the most association pattern, rather than evidence of fully independent effects. To address the dependence, we applied FDR correction to account for multiple comparisons, and evaluated the incremental value of NHR through C-statistic, category-free NRI, and IDI in regression models to assess whether NHR adds further discriminatory capacity without requiring the assumption of independence among indices.

Certainly, this study also has some limitations. First, the single-center retrospective design may limit the generalizability of the findings. Second, because the post-MT laboratory examinations were not mandatory, and were obtained at the discretion of physicians based on patient conditions, evaluating the associations between post-MT inflammatory-lipid indices and clinical outcomes may introduce substantial bias. Therefore, our study only analyzed inflammatory-lipid indices calculated from blood parameters at admission, which also precluded the exploration of the relationship between the dynamic trajectory of these indices and clinical outcomes. Third, although significant associations were observed, the AUC values of all inflammatory-lipid indices were modest, indicating limited discriminatory ability. Accordingly, these indices should not be regarded as standalone prognostic tools, and their clinical utility requires further validation in larger cohorts. Fourth, despite adjustment for a range of covariates in multivariable analysis, some unmeasured or unrecorded factors may still have influenced the results. The absence of these data may prevent us from fully elucidating the effect of inflammatory-lipid indices. Moreover, the selection of covariates in regression models was mainly based on significance in univariate analysis, which may result in the neglect of potential factors with meaningful associations with outcomes. Although we performed a prespecified sensitivity analysis using clinically and biologically relevant covariates, other potential confounders may still not be fully captured. Internal validation indicated acceptable model overfitting, while variable selection conducted on the same dataset can still introduce optimism into model performance. Thus, external validation is required to verify our findings. Fifth, the sample size of this study was limited, and the results of subgroup analyses based on a relatively small number of cases should be regarded as exploratory and interpreted with caution. Sixth, regarding subgroup stratification, the 350-min onset-to-recanalization cutoff was selected partly based on pathophysiological considerations. Prolonged onset-to-recanalization time is associated with greater infarct core expansion, more severe BBB disruption, and increased risk of reperfusion injury (Finitsis et al., 2014; Camara et al., 2021). The cutoff also referred to the distribution of our cohort, as 350 min approximated the median onset-to-recanalization time. This may limit the generalizability of the subgroup analysis findings. Finally, as an observational study, the results only indicated statistical correlations, which should not be regarded as establishing clinical predictive utility or causal relationships. Larger prospective multicenter studies with external validation are needed to determine whether these inflammatory-lipid indices can be used as predictive biomarkers in clinical practice.

## 5 Conclusion

This study comprehensively evaluated the associations between six inflammatory-lipid indices and clinical outcomes after MT. Among these indices, NHR was the most consistently associated with HT, MCE, 90-day unfavorable outcome, and all-cause mortality. In the subgroup analysis of all-cause mortality, an interaction between NHR and TOAST classification was observed, indicating that this association appeared stronger in patients with cardioembolic stroke. HT and MCE appeared to statistically account for part of the association between NHR and unfavorable outcome. Nevertheless, given the modest discriminatory performance and the single-center retrospective design, NHR should be regarded as a complementary factor within a multifactorial risk stratification framework, rather than as a generalizable or standalone prognostic biomarker. Further multicenter prospective studies are required to validate our findings and elucidate the mechanisms linking NHR and mortality.

## Data Availability

The raw data supporting the conclusions of this article will be made available by the authors, without undue reservation.
