# Supplementary material for: Associations between inflammatory-lipid indices and clinical outcomes in ischemic stroke patients treated with mechanical thrombectomy
Source: Front Aging Neurosci. 2026 Oct 2;18:1919645. doi: 10.3389/fnagi.2026.1919645 (PMC13672604; doi:10.3389/fnagi.2026.1919645)
Supplement: Supplementary file 1 [file Data_Sheet_1.DOCX]

Supplementary Table S1. covariates included for establishing regression models.

| Clinical outcome | covariates |
| --- | --- |
| Hemorrhagic transformation | Sex, age, initial NIHSS score, baseline ASPECTS, onset to recanalization time, intravenous thrombolysis, occluded vessel region, collateral score, presence of HMCAS, successful recanalization, and glucose |
| Malignant cerebral edema | Sex, age, initial NIHSS score, baseline ASPECTS, history of hypertension, occluded vessel region, collateral score, presence of HMCAS, sodium, and glucose |
| Unfavorable outcome | Sex, age, initial NIHSS score, baseline ASPECTS, onset to recanalization time, history of hypertension, collateral score, presence of HMCAS, successful recanalization, total calcium, sodium, glucose, and TG |
| All-cause mortality | Sex, age, smoker, initial NIHSS score, baseline ASPECTS, occluded vessel region, systolic pressure, collateral score, presence of HMCAS, successful recanalization, sodium, and glucose |

NIHSS: National Institute of Health Stroke Scale; ASPECTS: Alberta Stroke Program Early CT Score; HMCAS: Hyperdense middle cerebral artery sign; TG: Triglyceride.

Supplementary Table S2. Internal validation of regression models for clinical outcomes.

| Clinical outcomes | Original C-statistic | Optimism-corrected C-statistic^e^ | Slope Optimism | Calibration slope | Calibration intercept |
| --- | --- | --- | --- | --- | --- |
| Hemorrhagic transformation ^a^ | 0.763 | 0.737 | 0.139 | 0.861 | -0.095 |
| Malignant cerebral edema ^b^ | 0.741 | 0.708 | 0.167 | 0.833 | -0.173 |
| Unfavorable outcome ^c^ | 0.800 | 0.774 | 0.139 | 0.861 | 0.017 |
| All-cause mortality ^d^ | 0.810 | 0.785 | 0.126 | 0.874 | -0.105 |

^a^ Adjusted for sex, age, initial NIHSS score, baseline ASPECTS, onset to recanalization time, intravenous thrombolysis, occluded vessel region, collateral score, presence of HMCAS, successful recanalization, and glucose.

^b^ Adjusted for sex, age, initial NIHSS score, baseline ASPECTS, history of hypertension, occluded vessel region, collateral score, presence of HMCAS, sodium, and glucose;

^c^ Adjusted for sex, age, initial NIHSS score, baseline ASPECTS, onset to recanalization time, history of hypertension, collateral score, presence of HMCAS, successful recanalization, total calcium, sodium, glucose, and TG;

^d^ Adjusted for sex, age, smoker, initial NIHSS score, baseline ASPECTS, occluded vessel region, systolic pressure, collateral score, presence of HMCAS, successful recanalization, sodium, and glucose.

NIHSS: National Institute of Health Stroke Scale; ASPECTS: Alberta Stroke Program Early CT Score; HMCAS: Hyperdense middle cerebral artery sign; TG: Triglyceride.

Supplementary Table S3. Regression models established based on clinical or biological relevance.

| Clinical outcome | covariates |
| --- | --- |
| Hemorrhagic transformation | Sex, age, initial NIHSS score, baseline ASPECTS, onset to recanalization time, intravenous thrombolysis, occluded vessel region, collateral score, presence of HMCAS, successful recanalization, antiplatelet at onset, anticoagulant at onset, and glucose. |
| Malignant cerebral edema | Sex, age, initial NIHSS score, baseline ASPECTS, history of hypertension, occluded vessel region, collateral score, presence of HMCAS, and glucose |
| Unfavorable outcome | Sex, age, initial NIHSS score, baseline ASPECTS, onset to recanalization time, history of hypertension, collateral score, presence of HMCAS, successful recanalization, glucose, and TG |
| All-cause mortality | Sex, age, initial NIHSS score, baseline ASPECTS, occluded vessel region, collateral score, presence of HMCAS, successful recanalization, and glucose. |

NIHSS: National Institute of Health Stroke Scale; ASPECTS: Alberta Stroke Program Early CT Score; HMCAS: Hyperdense middle cerebral artery sign; TG: Triglyceride.

Supplementary Table S4. Baseline characteristics of patients with and without malignant cerebral edema.

|  | Malignant cerebral edema | Non malignant cerebral edema | p value |
| --- | --- | --- | --- |
| **Patients, n (%)** | 116 (22.6) | 397 (77.4) | - |
| **Demographic** |  |  |  |
| Female, n (%) | 63 (54.3) | 168 (42.3) | **0.029** |
| Age (years) | 75 [68-81] | 71 [62-78] | **< 0.001** |
| Smoker, n (%) | 17 (14.7) | 85 (21.4) | 0.141 |
| Alcoholism, n (%) | 15 (12.9) | 70 (17.6) | 0.291 |
| **Medical history, n (%)** |  |  |  |
| Hypertension | 70 (60.3) | 179 (45.1) | **0.005** |
| Diabetes mellitus | 21 (18.1) | 88 (22.2) | 0.417 |
| Coronary heart disease | 8 (6.9) | 56 (14.1) | 0.056 |
| Atrial fibrillation | 57 (49.1) | 200 (50.4) | 0.897 |
| Rheumatic heart disease | 10 (8.6) | 32 (8.1) | 0.999 |
| Heart failure | 6 (5.2) | 28 (7.1) | 0.614 |
| Prior stroke | 16 (13.8) | 51 (12.8) | 0.913 |
| Antiplatelet at onset | 3 (2.6) | 21 (5.3) | 0.336 |
| Anticoagulant at onset | 13 (11.2) | 43 (10.8) | 1.000 |
| **Clinical and imaging characteristics** |  |  |  |
| Systolic pressure (mmhg) | 146 [126-169] | 144 [125-160] | 0.107 |
| Diastolic pressure (mmhg) | 84 [74-95] | 83 [75-93] | 0.426 |
| Intravenous thrombolysis, n (%) | 40 (34.5) | 145 (36.5) | 0.770 |
| Initial NIHSS score | 17 [13-23] | 14 [11-18] | **< 0.001** |
| Baseline ASPECTS | 8 [7-9] | 8 [8-9] | **< 0.001** |
| Present HMCAS, n (%) | 60 (51.7) | 132 (33.2) | **< 0.001** |
| Occluded vessel region, n (%) |  |  | **0.002** |
| ICA | 56 (48.3) | 127 (32.0) |  |
| MCA | 60 (51.7) | 270 (68.0) |  |
| TOAST classification, n (%) |  |  | 0.418 |
| LAA | 37 (31.9) | 150 (37.8) |  |
| Cardioembolic | 68 (58.6) | 219 (55.2) |  |
| Undetermined or others | 11 (9.5) | 28 (7.1) |  |
| Collateral score, n (%) |  |  | **< 0.001** |
| Grade 0 | 48 (41.4) | 88 (22.2) |  |
| Grade 1 | 43 (37.1) | 167 (42.1) |  |
| Grade 2 | 25 (21.6) | 142 (35.8) |  |
| Onset to recanalization time (min) | 365 [301-477] | 352 [276-449] | 0.142 |
| Stent implantation, n (%) | 25 (21.6) | 91 (22.9) | 0.854 |
| Successful recanalization, n (%) | 103 (88.8) | 368 (92.7) | 0.248 |
| **Laboratory findings** |  |  |  |
| Total calcium (mmol/L) | 2.24 [2.16-2.31] | 2.23 [2.14-2.32] | 0.549 |
| Sodium (mmol/L) | 138.65 [135.40-140.22] | 139.00 [137.20-140.90] | **0.023** |
| Potassium (mmol/L) | 3.87 [3.53-4.20] | 3.80 [3.50-4.05] | 0.173 |
| Chloride (mmol/L) | 104.30 [102.18-106.48] | 104.50 [102.50-106.70] | 0.807 |
| Glucose (mmol/L) | 8.30 [6.89-10.12] | 7.00 [6.10-9.00] | **< 0.001** |
| CRP (mg/L) | 5.58 [1.96-14.12] | 3.48 [1.16-10.10] | **0.003** |
| White blood cell, × 10^9^/L | 8.65 [7.27-10.83] | 8.60 [7.10-10.70] | 0.342 |
| Neutrophil, × 10^9^/L | 7.88 [5.78-10.26] | 6.72 [4.95-8.74] | **< 0.001** |
| Monocyte, × 10^9^/L | 0.44 [0.26-0.55] | 0.46 [0.32-0.61] | 0.129 |
| Lymphocyte, × 10^9^/L | 1.00 [0.66-1.33] | 1.22 [0.86-1.68] | **< 0.001** |
| Hemoglobin, g/L | 128.00 [114.75-139.00] | 127.00 [116.00-138.00] | 0.841 |
| Platelet, × 10^9^/L | 185.00 [139.50-219.00] | 151.00 [114.00-192.00] | **< 0.001** |
| PT (s) | 12.05 [11.17-13.00] | 12.00 [11.10-12.90] | 0.755 |
| TT (s) | 17.30 [16.40-18.40] | 17.40 [16.30-18.20] | 0.271 |
| APTT (s) | 27.50 [25.08-32.80] | 27.90 [25.40-31.60] | 0.989 |
| Fibrinogen (g/L) | 2.80 [2.36-3.22] | 2.90 [2.46-3.37] | 0.295 |
| INR | 1.02 [0.95-1.10] | 1.02 [0.96-1.09] | 0.805 |
| Albumin (g/L) | 39.65 [36.40-42.10] | 39.60 [37.20-42.00] | 0.558 |
| TC (mmol/L) | 4.47 [3.87-5.23] | 4.41 [3.75-5.13] | 0.628 |
| TG (mmol/L) | 1.21 [0.86-1.95] | 1.19 [0.90-1.74] | 0.787 |
| LDL-C (mmol/L) | 2.17 [1.66-2.77] | 2.18 [1.65-2.64] | 0.944 |
| HDL-C (mmol/L) | 1.24 [1.03-1.48] | 1.32 [1.13-1.51] | 0.080 |

Bold values indicate statistical significance.

NIHSS: National Institute of Health Stroke Scale; ASPECTS: Alberta Stroke Program Early CT Score; HMCAS: Hyperdense middle cerebral artery sign; ICA: Internal carotid artery; MCA: Middle cerebral artery; TOAST: Trial of ORG 10172 in Acute Stroke Treatment; LAA: Large-artery atherosclerosis; CRP: C-reactive protein; PT: Prothrombin time; TT: Thrombin time; APTT: Activated partial thromboplastin time; INR: International normalized ratio; TC: Total cholesterol; TG: Triglyceride; HDL-C: High-density lipoprotein cholesterol; LDL-C: Low-density lipoprotein cholesterol;

Supplementary Table S5. Baseline characteristics of patients with 90-day unfavorable and favorable outcome.

|  | Unfavorable outcome | Favorable outcome | p value |
| --- | --- | --- | --- |
| **Patients, n (%)** | 281 (54.8) | 232 (45.2) | - |
| **Demographic** |  |  |  |
| Female, n (%) | 136 (48.4) | 95 (40.9) | 0.110 |
| Age (years) | 74 [67-80] | 70 [59-77] | **< 0.001** |
| Smoker, n (%) | 53 (18.9) | 49 (21.1) | 0.598 |
| Alcoholism, n (%) | 43 (15.3) | 42 (18.1) | 0.465 |
| **Medical history, n (%)** |  |  |  |
| Hypertension | 148 (52.7) | 101 (43.5) | **0.049** |
| Diabetes mellitus | 66 (23.5) | 43 (18.5) | 0.209 |
| Coronary heart disease | 38 (13.5) | 26 (11.2) | 0.512 |
| Atrial fibrillation | 150 (53.4) | 107 (46.1) | 0.122 |
| Rheumatic heart disease | 21 (7.5) | 21 (9.1) | 0.626 |
| Heart failure | 13 (4.6) | 21 (9.1) | 0.068 |
| Prior stroke | 42 (14.9) | 25 (10.8) | 0.206 |
| Antiplatelet at onset | 15 (5.3) | 9 (3.9) | 0.570 |
| Anticoagulant at onset | 31 (11.0) | 25 (10.8) | 1.000 |
| **Clinical and imaging characteristics** |  |  |  |
| Systolic pressure (mmhg) | 145 [125-164] | 144 [125-160] | 0.564 |
| Diastolic pressure (mmhg) | 83 [74-93] | 83 [75-94] | 0.918 |
| Intravenous thrombolysis, n (%) | 97 (34.5) | 88 (37.9) | 0.479 |
| Initial NIHSS score | 16 [13-20] | 13 [10-16] | **< 0.001** |
| Baseline ASPECTS | 8 [7-8] | 8 [8-9] | **< 0.001** |
| Present HMCAS, n (%) | 122 (43.4) | 70 (30.2) | **0.003** |
| Occluded vessel region, n (%) |  |  | 0.057 |
| ICA | 111 (39.5) | 72 (31.0) |  |
| MCA | 170 (60.5) | 160 (69.0) |  |
| TOAST classification, n (%) |  |  | 0.495 |
| LAA | 96 (34.2) | 91 (39.2) |  |
| Cardioembolic | 163 (58.0) | 124 (53.4) |  |
| Undetermined or others | 22 (7.8) | 17 (7.3) |  |
| Collateral score, n (%) |  |  | **< 0.001** |
| Grade 0 | 106 (37.7) | 30 (12.9) |  |
| Grade 1 | 105 (37.4) | 105 (45.3) |  |
| Grade 2 | 70 (24.9) | 97 (41.8) |  |
| Onset to recanalization time (min) | 373 [304-477] | 330 [259-421] | **< 0.001** |
| Stent implantation, n (%) | 61 (21.7) | 55 (23.7) | 0.665 |
| Successful recanalization, n (%) | 248 (88.3) | 223 (96.1) | **0.002** |
| **Laboratory findings** |  |  |  |
| Total calcium (mmol/L) | 2.25 [2.16-2.33] | 2.22 [2.14-2.29] | **0.006** |
| Sodium (mmol/L) | 138.90 [136.50-140.60] | 139.20 [137.38-141.30] | **0.006** |
| Potassium (mmol/L) | 3.80 [3.50-4.10] | 3.80 [3.50-4.02] | 0.198 |
| Chloride (mmol/L) | 104.20 [102.30-106.40] | 104.95 [102.85-106.90] | 0.133 |
| Glucose (mmol/L) | 7.90 [6.70-10.00] | 6.80 [5.80-8.40] | **< 0.001** |
| CRP (mg/L) | 5.50 [2.02-14.10] | 2.64 [0.94-6.90] | **< 0.001** |
| White blood cell, × 10^9^/L | 8.80 [7.40-11.30] | 8.60 [6.80-10.40] | **0.019** |
| Neutrophil, × 10^9^/L | 7.50 [5.67-9.91] | 6.32 [4.70-8.16] | **< 0.001** |
| Monocyte, × 10^9^/L | 0.45 [0.30-0.61] | 0.46 [0.32-0.60] | 0.637 |
| Lymphocyte, × 10^9^/L | 1.02 [0.69-1.47] | 1.30 [0.99-1.83] | **< 0.001** |
| Hemoglobin, g/L | 126.00 [114.00-137.00] | 128.00 [117.00-139.00] | 0.191 |
| Platelet, × 10^9^/L | 166.00 [124.00-209.00] | 147.00 [113.00-188.25] | **0.002** |
| PT (s) | 12.10 [11.20-13.00] | 11.75 [11.00-12.83] | 0.056 |
| TT (s) | 17.30 [16.30-18.10] | 17.40 [16.40-18.40] | 0.513 |
| APTT (s) | 27.80 [25.40-32.60] | 27.90 [25.10-31.30] | 0.219 |
| Fibrinogen (g/L) | 2.90 [2.49-3.34] | 2.88 [2.38-3.41] | 0.607 |
| INR | 1.02 [0.96-1.10] | 1.01 [0.96-1.09] | 0.419 |
| Albumin (g/L) | 39.50 [36.90-42.10] | 39.70 [37.38-42.30] | 0.283 |
| TC (mmol/L) | 4.37 [3.86-5.11] | 4.46 [3.75-5.16] | 0.573 |
| TG (mmol/L) | 1.29 [0.90-1.93] | 1.13 [0.86-1.61] | **0.016** |
| LDL-C (mmol/L) | 2.18 [1.66-2.77] | 2.15 [1.65-2.58] | 0.304 |
| HDL-C (mmol/L) | 1.26 [1.05-1.45] | 1.35 [1.17-1.58] | **< 0.001** |

Bold values indicate statistical significance.

NIHSS: National Institute of Health Stroke Scale; ASPECTS: Alberta Stroke Program Early CT Score; HMCAS: Hyperdense middle cerebral artery sign; ICA: Internal carotid artery; MCA: Middle cerebral artery; TOAST: Trial of ORG 10172 in Acute Stroke Treatment; LAA: Large-artery atherosclerosis; CRP: C-reactive protein; PT: Prothrombin time; TT: Thrombin time; APTT: Activated partial thromboplastin time; INR: International normalized ratio; TC: Total cholesterol; TG: Triglyceride; HDL-C: High-density lipoprotein cholesterol; LDL-C: Low-density lipoprotein cholesterol;

Supplementary Table S6. Baseline characteristics of patients with and without 90-day all-cause mortality

|  | Mortality | Survival | HR (95% CI) | p value* |
| --- | --- | --- | --- | --- |
| **Patients, n (%)** | 133 (25.9) | 380 (74.1) | - | - |
| **Demographic** |  |  |  |  |
| Female, n (%) | 75 (56.4) | 156 (41.1) | 1.771 (1.257-2.495) | **0.001** |
| Age (years) | 76 [69-82] | 71 [61-77] | 1.043 (1.026-1.061) | **< 0.001** |
| Smoker, n (%) | 18 (13.5) | 84 (22.1) | 0.569 (0.346-0.935) | **0.026** |
| Alcoholism, n (%) | 20 (15.0) | 65 (17.1) | 0.821 (0.510-1.321) | 0.416 |
| **Medical history, n (%)** |  |  |  |  |
| Hypertension | 72 (54.1) | 177 (46.6) | 1.286 (0.914-1.809) | 0.148 |
| Diabetes mellitus | 29 (21.8) | 80 (21.1) | 1.012 (0.671-1.528) | 0.953 |
| Coronary heart disease | 18 (13.5) | 46 (12.1) | 1.079 (0.657-1.774) | 0.764 |
| Atrial fibrillation | 67 (50.4) | 190 (50.0) | 1.021 (0.727-1.434) | 0.906 |
| Rheumatic heart disease | 11 (8.3) | 31 (8.2) | 1.031 (0.556-1.910) | 0.923 |
| Heart failure | 7 (5.3) | 27 (7.1) | 0.720 (0.336-1.541) | 0.397 |
| Prior stroke | 18 (13.5) | 49 (12.9) | 1.031 (0.627-1.695) | 0.904 |
| Antiplatelet at onset | 6 (4.5) | 18 (4.7) | 0.937 (0.413-2.126) | 0.877 |
| Anticoagulant at onset | 14 (10.5) | 42 (11.1) | 0.930 (0.535-1.618) | 0.797 |
| **Clinical and imaging characteristics** |  |  |  |  |
| Systolic pressure (mmhg) | 152 [139-168] | 144 [124-160] | 1.009 (1.003-1.014) | **0.004** |
| Diastolic pressure (mmhg) | 85 [78-95] | 83 [74-93] | 1.009 (1.000-1.019) | 0.053 |
| Intravenous thrombolysis, n (%) | 48 (36.1) | 137 (36.1) | 1.002 (0.704-1.428) | 0.990 |
| Initial NIHSS score | 18 [14-23] | 14 [11-18] | 1.084 (1.058-1.110) | **< 0.001** |
| Baseline ASPECTS | 7 [7-8] | 8 [8-9] | 0.635 (0.555-0.726) | **< 0.001** |
| Present HMCAS, n (%) | 73 (54.9) | 119 (31.3) | 2.432 (1.728-3.423) | **< 0.001** |
| Occluded vessel region, n (%) |  |  |  |  |
| MCA | 65 (48.9) | 265 (69.7) | Reference |  |
| ICA | 68 (51.1) | 115 (30.3) | 2.187 (1.556-3.073) | **< 0.001** |
| TOAST classification, n (%) |  |  |  |  |
| Cardioembolic | 79 (59.4) | 208 (54.7) | Reference |  |
| LAA | 42 (31.6) | 145 (38.2) | 0.769 (0.529-1.118) | 0.170 |
| Undetermined or others | 12 (9.0) | 27 (7.1) | 1.120 (0.610-2.056) | 0.714 |
| Collateral score, n (%) |  |  |  |  |
| Grade 0 | 66 (49.6) | 70 (18.4) | Reference |  |
| Grade 1 | 43 (32.3) | 167 (43.9) | 0.337 (0.229-0.495) | **< 0.001** |
| Grade 2 | 24 (18.0) | 143 (37.6) | 0.230 (0.144-0.368) | **< 0.001** |
| Onset to recanalization time (min) | 359 [297-447] | 352 [275-458] | 1.000 (0.999-1.001) | 0.641 |
| Stent implantation, n (%) | 26 (19.5) | 90 (23.7) | 0.806 (0.525-1.237) | 0.323 |
| Successful recanalization, n (%) | 109 (82.0) | 362 (95.3) | 0.280 (0.180-0.436) | **< 0.001** |
| **Laboratory findings** |  |  |  |  |
| Total calcium (mmol/L) | 2.24 [2.14-2.32] | 2.23 [2.14-2.31] | 1.194 (0.377-3.782) | 0.763 |
| Sodium (mmol/L) | 138.50 [135.00-140.30] | 139.00 [137.20-141.00] | 0.928 (0.890-0.967) | **< 0.001** |
| Potassium (mmol/L) | 3.80 [3.44-4.16] | 3.80 [3.51-4.06] | 0.974 (0.659-1.439) | 0.895 |
| Chloride (mmol/L) | 104.20 [102.00-106.40] | 104.65 [102.70-106.82] | 0.980 (0.938-1.024) | 0.362 |
| Glucose (mmol/L) | 8.40 [6.80-10.20] | 7.00 [6.10-8.90] | 1.089 (1.049-1.130) | **< 0.001** |
| CRP (mg/L) | 5.50 [2.02-14.13] | 3.28 [1.17-9.62] | 1.005 (0.997-1.013) | 0.231 |
| White blood cell, × 10^9^/L | 9.50 [7.50-12.10] | 8.60 [7.07-10.60] | 1.047 (0.996-1.101) | 0.074 |
| Neutrophil, × 10^9^/L | 7.92 [5.99-10.17] | 6.64 [4.94-8.66] | 1.104 (1.056-1.154) | **< 0.001** |
| Monocyte, × 10^9^/L | 0.45 [0.32-0.59] | 0.45 [0.32-0.61] | 0.968 (0.479-1.957) | 0.928 |
| Lymphocyte, × 10^9^/L | 0.92 [0.69-1.27] | 1.26 [0.88-1.73] | 0.422 (0.303-0.589) | **< 0.001** |
| Hemoglobin, g/L | 126.00 [113.00-137.00] | 127.50 [116.00-138.25] | 0.996 (0.987-1.004) | 0.332 |
| Platelet, × 10^9^/L | 186.00 [137.00-222.00] | 151.00 [114.00-190.00] | 1.004 (1.002-1.006) | **< 0.001** |
| PT (s) | 12.10 [11.20-13.00] | 11.90 [11.10-12.90] | 0.976 (0.901-1.058) | 0.554 |
| TT (s) | 17.30 [16.10-18.20] | 17.40 [16.40-18.30] | 0.985 (0.942-1.030) | 0.507 |
| APTT (s) | 27.70 [24.90-32.10] | 28.00 [25.48-31.60] | 0.988 (0.963-1.014) | 0.366 |
| Fibrinogen (g/L) | 2.90 [2.43-3.32] | 2.88 [2.41-3.36] | 0.933 (0.782-1.113) | 0.438 |
| INR | 1.04 [0.96-1.10] | 1.01 [0.96-1.09] | 0.647 (0.245-1.704) | 0.378 |
| Albumin (g/L) | 39.70 [37.00-42.10] | 39.55 [37.10-42.10] | 0.988 (0.950-1.028) | 0.550 |
| TC (mmol/L) | 4.47 [3.86-5.11] | 4.40 [3.76-5.14] | 0.977 (0.843-1.133) | 0.762 |
| TG (mmol/L) | 1.25 [0.88-1.94] | 1.19 [0.87-1.74] | 1.099 (0.969-1.246) | 0.143 |
| LDL-C (mmol/L) | 2.16 [1.66-2.86] | 2.18 [1.65-2.62] | 1.091 (0.883-1.347) | 0.419 |
| HDL-C (mmol/L) | 1.30 [1.09-1.46] | 1.32 [1.12-1.54] | 0.661 (0.412-1.059) | 0.085 |

*Indicating the results of univariate Cox regression analysis.

Bold values indicate statistical significance.

NIHSS: National Institute of Health Stroke Scale; ASPECTS: Alberta Stroke Program Early CT Score; HMCAS: Hyperdense middle cerebral artery sign; ICA: Internal carotid artery; MCA: Middle cerebral artery; TOAST: Trial of ORG 10172 in Acute Stroke Treatment; LAA: Large-artery atherosclerosis; CRP: C-reactive protein; PT: Prothrombin time; TT: Thrombin time; APTT: Activated partial thromboplastin time; INR: International normalized ratio; TC: Total cholesterol; TG: Triglyceride; HDL-C: High-density lipoprotein cholesterol; LDL-C: Low-density lipoprotein cholesterol; HR: Hazard ratio; CI: Confidence interval.

Supplementary Table S7. Sensitivity analysis revealing the associations between inflammatory-lipid indices and clinical outcomes after adjusting for clinically/biologically relevant covariates.

| Inflammatory-lipid indices | Hemorrhagic transformation ^a^ | | | Malignant cerebral edema ^b^ | | | Unfavorable outcome ^c^ | | | All-cause mortality ^d^ | | |
| --- | --- | --- | --- | --- | --- | --- | --- | --- | --- | --- | --- | --- |
|  | OR (95% CI) | p value | p-FDR value | OR (95% CI) | p value | p-FDR value | OR (95% CI) | p value | p-FDR value | HR (95% CI) | p value | p-FDR value |
| MHR | 0.802 (0.338-1.882) | 0.613 | 0.613 | 0.758 (0.302-1.781) | 0.537 | 0.645 | 2.493 (1.031-6.296) | 0.047 | 0.056 | 0.913 (0.463-1.802) | 0.795 | 0.876 |
| PHR* | 1.282 (0.953-1.726) | 0.098 | 0.147 | 1.710 (1.263-2.324) | < 0.001 | 0.003 | 1.738 (1.275-2.413) | < 0.001 | 0.001 | 1.373 (1.122-1.680) | 0.002 | 0.012 |
| NHR | 1.079 (1.023-1.143) | 0.006 | 0.038 | 1.089 (1.031-1.154) | 0.002 | 0.008 | 1.172 (1.095-1.261) | < 0.001 | < 0.001 | 1.047 (1.012-1.082) | 0.007 | 0.014 |
| LHR | 0.665 (0.465-0.931) | 0.020 | 0.062 | 0.795 (0.547-1.120) | 0.209 | 0.313 | 0.960 (0.704-1.308) | 0.799 | 0.799 | 0.625 (0.445-0.880) | 0.007 | 0.014 |
| FHR | 0.951 (0.790-1.132) | 0.590 | 0.613 | 0.993 (0.822-1.184) | 0.946 | 0.946 | 1.294 (1.082-1.564) | 0.005 | 0.011 | 0.964 (0.835-1.114) | 0.625 | 0.876 |
| CRP/HDL-C | 1.013 (0.999-1.027) | 0.059 | 0.119 | 1.013 (1.000-1.027) | 0.044 | 0.089 | 1.023 (1.006-1.044) | 0.011 | 0.017 | 0.999 (0.990-1.008) | 0.876 | 0.876 |

* Per 100 units

^a^ Adjusted for sex, age, initial NIHSS score, baseline ASPECTS, onset to recanalization time, intravenous thrombolysis, occluded vessel region, collateral score, presence of HMCAS, successful recanalization, antiplatelet at onset, anticoagulant at onset, and glucose.

^b^ Adjusted for sex, age, initial NIHSS score, baseline ASPECTS, history of hypertension, occluded vessel region, collateral score, presence of HMCAS, and glucose;

^c^ Adjusted for sex, age, initial NIHSS score, baseline ASPECTS, onset to recanalization time, history of hypertension, collateral score, presence of HMCAS, successful recanalization, glucose, and TG;

^d^ Adjusted for sex, age, initial NIHSS score, baseline ASPECTS, occluded vessel region, collateral score, presence of HMCAS, successful recanalization, and glucose.

MHR: monocyte-to-HDL-C ratio; PHR: platelet-to-HDL-C ratio; NHR: neutrophil-to-HDL-C ratio; LHR: lymphocyte-to-HDL-C ratio; FHR: fibrinogen-to-HDL-C ratio; CRP/HDL-C: C-reactive protein-to-HDL-C ratio; HDL-C: High-density lipoprotein cholesterol; OR: Odds ratio; HR: Hazard ratio; CI: Confidence interval; NIHSS: National Institute of Health Stroke Scale; ASPECTS: Alberta Stroke Program Early CT Score; HMCAS: Hyperdense middle cerebral artery sign; TG: Triglyceride.

Supplementary Table S8. False discovery rate (FDR) correction for the results of multiple interaction tests in subgroup analysis.

| Subgroups | Hemorrhagic transformation | | Malignant cerebral edema | | Unfavorable outcome | | All-cause mortality | |
| --- | --- | --- | --- | --- | --- | --- | --- | --- |
|  | p for interaction | p-FDR for interaction | p for interaction | p-FDR for interaction | p for interaction | p-FDR for interaction | p for interaction | p-FDR for interaction |
| Sex | 0.573 | 0.611 | 0.683 | 0.769 | 0.293 | 0.606 | 0.284 | 0.445 |
| Age | 0.611 | 0.611 | 0.403 | 0.705 | 0.433 | 0.606 | 0.136 | 0.317 |
| Intravenous thrombolysis | 0.546 | 0.611 | 0.769 | 0.769 | 0.288 | 0.606 | 0.396 | 0.462 |
| Occluded vessel | 0.292 | 0.611 | 0.559 | 0.769 | 0.587 | 0.684 | 0.554 | 0.554 |
| TOAST classification | 0.364 | 0.611 | 0.101 | 0.235 | 0.808 | 0.808 | 0.023 | 0.161 |
| Onset to recanalization time | 0.112 | 0.549 | 0.076 | 0.235 | 0.365 | 0.606 | 0.067 | 0.234 |
| Collateral score | 0.157 | 0.549 | 0.101 | 0.235 | 0.339 | 0.606 | 0.318 | 0.445 |

TOAST: Trial of ORG 10172 in Acute Stroke Treatment
